# Gold-Catalyzed Propargylic Substitution Followed by Cycloisomerization in Ionic Liquid: Environmentally Friendly Synthesis of Polysubstituted Furans from Propargylic Alcohols and 1,3-Dicarbonyl Compounds

**DOI:** 10.3390/molecules29225441

**Published:** 2024-11-18

**Authors:** Hitomi Chiaki, Yoshimitsu Hashimoto, Nobuyoshi Morita

**Affiliations:** Department of Pharmacy, Showa Pharmaceutical University, Machida 194-8543, Japan

**Keywords:** gold catalyst, ionic liquid, propargylic substitution, cycloisomerization, furans

## Abstract

Gold-catalyzed propargylic substitution of propargylic alcohols **1** with 1,3-dicarbonyl compounds **2** followed by cycloisomerization in ionic liquid enables the environmentally friendly synthesis of polysubstituted furans **3** in good-to-high yields. The reaction proceeds via the hydrated propargylic substitution product **3″aa**. The gold catalyst can be recycled at least three times.

## 1. Introduction

**Scheme 1:**
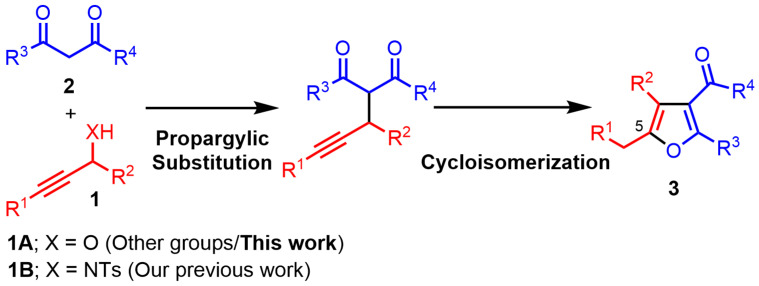
Propargylic substitution followed by cycloisomerization for synthesis of polysubstituted furan.

Polysubstituted furans are frequently found as structural components of natural products [1] and biologically active compounds [2,3,4] and are also useful as reagents and intermediates [5,6] in organic synthesis. Therefore, various synthetic methods have been developed by many chemists over a long period of time [7,8,9]. One of many procedures for the synthesis of polysubstituted furans involves the powerful approaches of transition-metal-catalyzed propargylic substitution followed by cycloisomerization (Scheme 1). 

For example, the efficient synthesis of polysubstituted furans **3** was achieved via a propargylic substitution/cycloisomerization sequence from propargylic alcohols **1A** and 1,3-dicarbonyl compounds **2** using a combination of transition metals and reagents (Ag/Cs_2_CO_3_ [10], Ir-Sn/Cs_2_CO_3_ [11]). On the other hand, Arcadi reported the gold-catalyzed propargylic substitution [12] of propargylic alcohol **1A** followed by cycloisomerization to produce polysubstituted furans **3** [13]. We also reported the synthesis of polysubstituted furans **3** via propargylic substitution/cycloisomerization sequence from propargylic amides **1B** using a gold catalyst [14]. However, these methodologies using a transition metal catalyst/reagent combination [10,11] and gold catalysts [13,14] require toxic halogenated or volatile organic solvents, making it essential to avoid the use of organic solvents and to reuse catalysts to reduce environmental impact and develop sustainable chemical synthesis methods.

One of the ways to achieve sustainable chemical synthesis [15,16] is to use ionic liquids instead of organic solvents [17,18,19,20,21,22,23,24,25,26]. Ionic liquids have the following features: (1) non-volatility; (2) high solubility in many organic compounds as well as transition metal catalysts; (3) easy extraction of the products in ionic liquids with non-polar organic solvents; and (4) recyclability of transition metal catalysts in ionic liquids. For these reasons, ionic liquids are used as an alternative solvent to organic solvents.

Recently, we developed the environmentally friendly and stereoselective synthesis of 1,2,3-trisubstituted indanes and 2,3-dihydrobenzofurans by gold(I)/(III) catalysts in ionic liquid [27,28,29] or water [30]. In these methods, environmentally friendly ionic liquid or water is used as a solvent, gold-catalyzed reactions for synthesis of cyclic compounds are achieved, and the gold catalysts in the ionic liquids are successfully reused.

Continuing our research of sustainable chemical synthesis methods, we focused on the gold-catalyzed synthesis of polysubstituted furans in ionic liquid [17,18]. Herein, we present a gold-catalyzed propargylic substitution with 1,3-dicarbonyl compounds followed by cycloisomerization for the environmentally friendly synthesis of polysubstituted furans. The advantages of this approach from an environmental and economic point of view are that it does not use toxic or volatile solvents, does not require stoichiometric amounts of reagents, and allows the gold catalyst in the ionic liquid to be reused several times.

## 2. Results and Discussion

We first examined propargylic substitution of propargylic alcohol **1a** with acetylacetone (**2a**) followed by cycloisomerization in the presence of an oxophilic gold(III) catalyst to activate the hydroxyl group at the propargylic position (Table 1). Treatment of propargylic alcohol **1a** with acetylacetone (**2a**) in the presence of gold(III) catalyst (5 mol% AuBr_3_) at room temperature for 3 days in an ionic liquid, [ethylmethylimidazolium (EMIM)][NTf_2_], afforded the desired furan **3aa** in low yield (entry 1). The yield of **3aa** was improved to 72% yield when the reaction was carried out with the addition of a silver catalyst (15 mol% AgOTf) to activate the gold catalyst (entry 2). In an attempt to further improve the yield, the reaction was carried out at 100 °C for 30 min, but the yield of the desired furan **3aa** decreased (entry 3). However, reducing the reaction temperature from 100 °C to 60 °C afforded **3aa** in 84% yield (entry 4). When the amount of acetylacetone (**2a**) was increased from 1 eq. to 3 eq., the yield of the desired furan **3aa** decreased slightly (entry 5). In contrast to the reaction using gold(III) catalyst, the reaction in the presence of gold(I) catalyst (5 mol% AuCl or 5 mol% AuCl with 5 mol% AgOTf) was less effective (entries 6 and 7).

Next, the effect of the counter anion of the silver catalyst (AgNTf_2_, AgBF_4_, AgSbF_6_) was investigated (Table 2). Treatment of propargylic alcohol **1a** with acetylacetone (**2a**) in the presence of AuBr_3_ (5 mol%) with AgNTf_2_ (15 mol%) or AgBF_4_ (15 mol%) in [EMIM][NTf_2_] at 60 °C afforded the corresponding furan **3aa** in moderate yield, 57% or 47%, respectively (entries 2 and 3). The use of AgSbF_6_ (15 mol%) in the reaction failed to generate the desired product **3aa** (entry 4). 

To examine the effect of the ionic liquid, we conducted reactions with [EMIM][CH_3_CO_2_], [EMIM][Me_2_PO_4_], [EMIM][HSO_4_], [EMIM][MeSO_4_], and [EMIM][OTf] (Table 3). With [EMIM][CH_3_CO_2_] or [EMIM][Me_2_PO_4_], the reaction did not proceed at all (entries 2 and 3). [EMIM][HSO_4_], [EMIM][MeSO_4_] and [EMIM][OTf] afforded trace amounts of the desired furan **3aa** (entries 4–6). The use of an organic solvent, dichloroethane (ClCH_2_CH_2_Cl), instead of [EMIM][NTf_2_] reduced the yield of **3aa** (entry 7). These experiments showed that [EMIM][NTf_2_] is the preferred solvent for this transformation.

Lee’s group reported various advantages of ionic liquids for catalytic reactions, including the formation of more reactive catalysts and the stabilization of reactive intermediate and transition states [31]. The exact reason for the activity of AuBr_3_ and AgOTf in ionic liquids is still unclear, but two possibilities related to the formation of the active gold species, Au(NTf_2_)_3_ and NHC-Au species (NHC = *N*-Heterocyclic Carbene ligand) can be considered. The first possibility is the formation of an active gold species, Au(NTf_2_)_3_, which is generated from the exchange reaction between the chloride ion of the gold catalyst and the NTf_2_ ion of the ionic liquid, [EMIM][NTf_2_]. The second possibility is the formation of an active NHC–gold species. This active species might be generated from the EMIM cation of [EMIM][NTf_2_]. The possibility for the generation of this type of active species has been reported by other groups [32,33] and might also be possible with gold catalysts.

**Figure 1:**
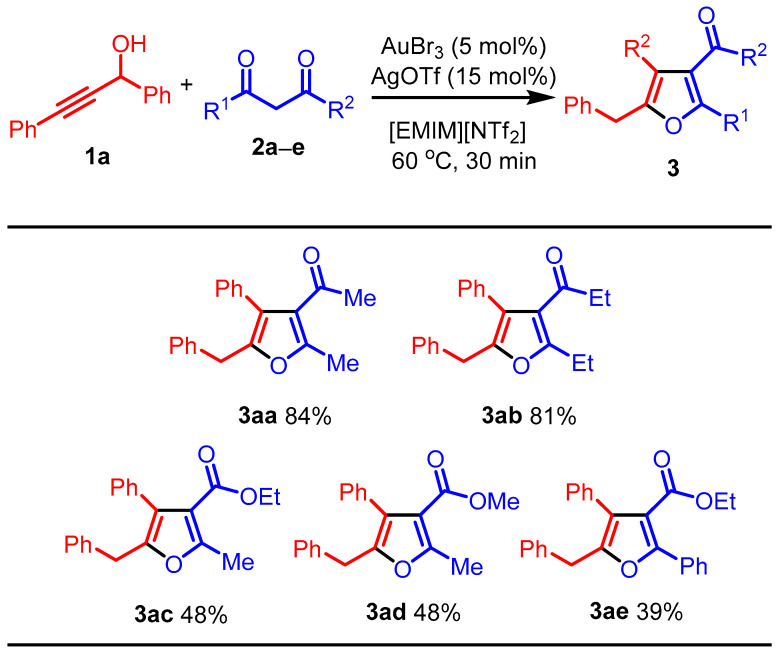
Scope and limitations of gold-catalyzed propargylic substitution of propargylic alcohol **1a** with 1,3-dicarbonyl compounds **2a**–**e** followed by cycloisomerization.

We next investigated the scope and limitations of this procedure for the synthesis of polysubstituted furans **3** by using propargylic alcohol **1a** with various 1,3-dicarbonyl compounds **2a**–**e** in the presence of 5 mol% AuBr_3_ and 15 mol% AgOTf in [EMIM][NTf_2_] (Figure 1). Various combinations of propargylic alcohol **1a** with 1,3-dicarbonyl compounds **2a**–**e** afforded the corresponding products **3** in good to high yields. The reaction proceeded not only with 1,3-diketones **2a** and **2b**, but also with β-ketoesters **2c**–**e**, although the yield of furan **3** was somewhat lower with β-ketoesters **2c**–**e**.

**Scheme 2:**
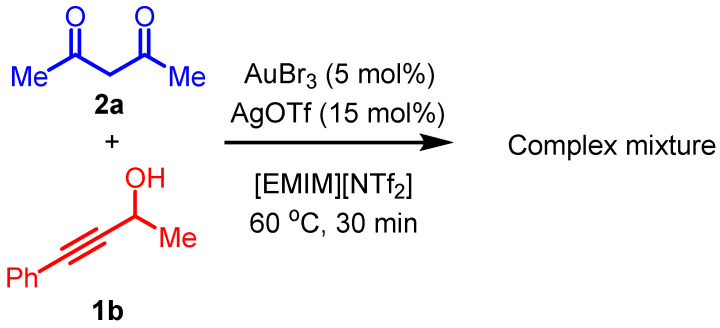
The reaction of propargylic alcohol **1b** with acetylacetone (**2a**).

To investigate the scope and limitations of this procedure, we tried the reaction of propargylic alcohol **1b** bearing a methyl group at the propargylic position instead of a phenyl group. However, the reaction with **1b** afforded a complex mixture (Scheme 2). This result suggests that the phenyl group at the propargylic position is essential for the formation of polysubstituted furans **3**. 

**Scheme 3:**
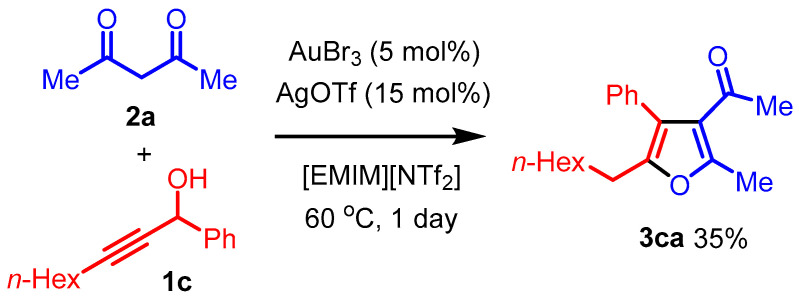
The reaction of propargylic alcohol **1c** with acetylacetone (**2a**).

Next, we attempted the reaction of propargylic alcohol **1c** bearing a hexyl group at the terminus of acetylene instead of a phenyl group. The reaction of **1c** resulted in a low yield of the corresponding polysubstituted furan **3ca** (Scheme 3).

**Scheme 4:**
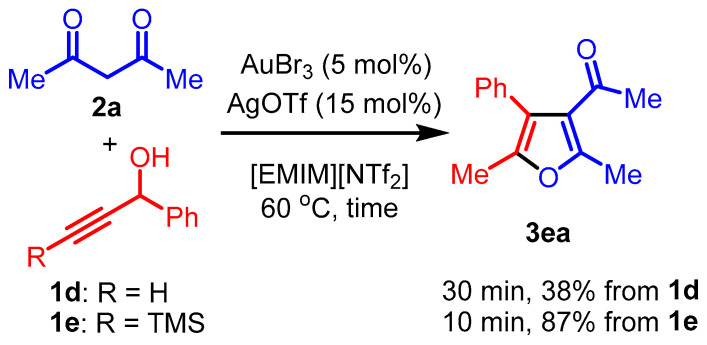
The reaction of propargylic alcohol **1d** (R = H) and **1e** (R = TMS) with acetylacetone (**2a**).

We next investigated the reaction of propargylic alcohol **1d** without substituent (R = H) on the terminal of acetylene (Scheme 4). Treatment of **1d** with acetylacetone (**2a**) in the presence of AuBr_3_ (5 mol%) and AgOTf (15 mol%) in [EMIM][NTf_2_] at 60 °C for 30 min afforded the corresponding furan **3ea** in 38% yield. On the other hand, the reaction with propargylic alcohol **1e** bearing a trimethylsilyl group (R = TMS) gave the product **3ea** in high yield, with desilylation (Scheme 4). Additionally, it was observed that the reaction was faster for substrates with silyl group than for those with phenyl group (reaction time; **1a**: 30 min/**1e**: 10 min). This is probably due to the β-cation-stabilizing effect [34,35] of the silyl group, which would promote cycloisomerization (see Scheme 7). This phenomenon was also observed in our previous work [14].

**Figure 2:**
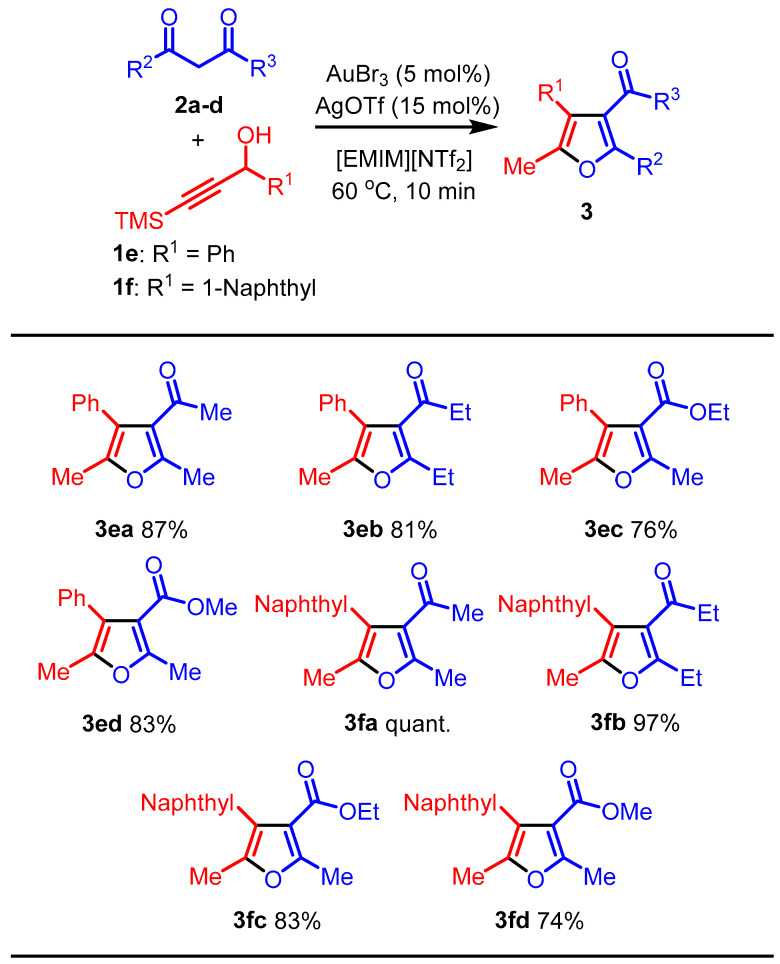
Scope and limitations of gold-catalyzed propargylic substitution of propargylic alcohols **1e** and **1f** with 1,3-dicarbonyl compounds **2a**–**d** followed by cycloisomerization.

Next, we investigated the scope and limitations of this procedure for the synthesis of polysubstituted furans **3** by using propargylic alcohol **1e** and **1f** bearing a silyl group at the terminal position of acetylene with various 1,3-dicarbonyl compounds **2a**–**e** in the presence of 5 mol% AuBr_3_ and 15 mol% AgOTf in [EMIM][NTf_2_] (Figure 2). The reaction proceeded smoothly to give the corresponding furans **3** in good to high yields. In addition, the reaction was found to be faster than with a phenyl group at the terminus of acetylene for all substrate combinations; the reaction proceeded within 10 min. 

**Scheme 5:**
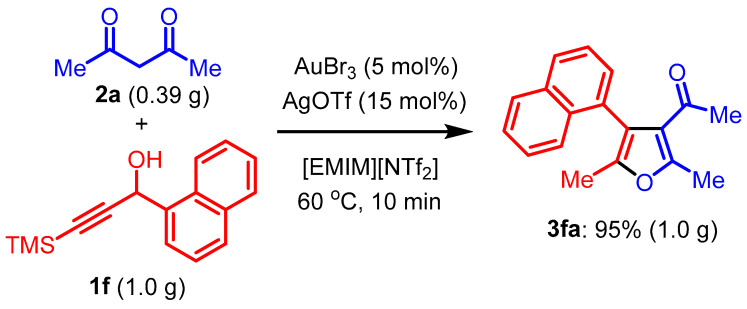
Large-scale preparation of polysubstituted furan **3fa** from propargylic alcohol **1f** with acetylacetone (**2a**).

Next, a large-scale preparation of polysubstituted furan **3fa** was conducted (Scheme 5), using 1.0 g (3.9 mmol) of propargylic alcohol **1f** and acetylacetone (**2a**), affording the corresponding product **3fa** in 95% yield. 

The reuse of the gold catalyst in ionic liquid is of great importance from both an environmental and economic point of view. Fortunately, the gold catalyst (5 mol% of AuBr_3_ 15 mol% of AgOTf/ionic liquid [EMIM][NTf_2_]) could be recycled at least three times for the reaction of **1f** with acetylacetone (**2a**) with only slight loss of activity (Table 4).

**Scheme 6:**
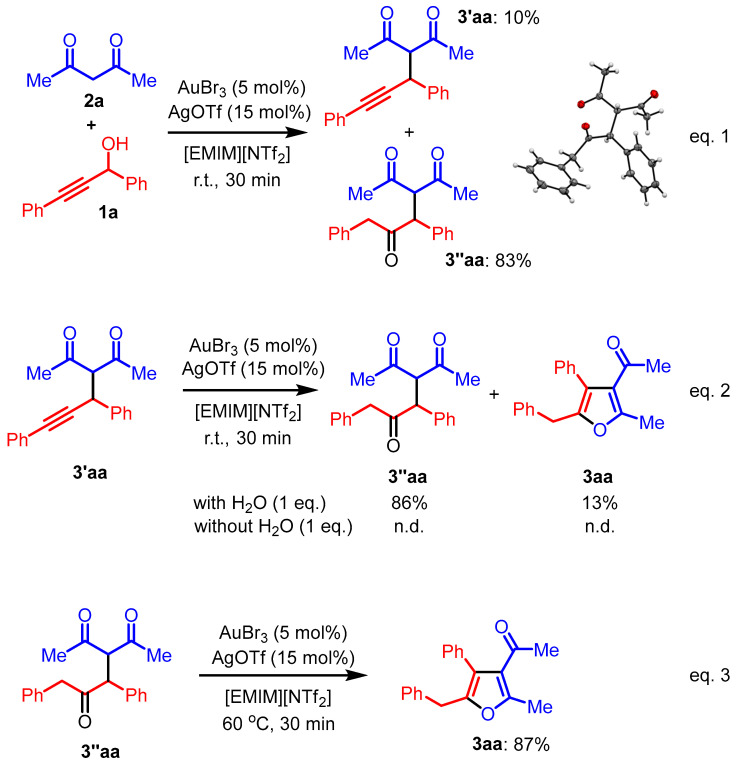
Generation of intermediate **3′aa** leading to furan **3aa**.

To confirm the reaction pathway, we examined the reaction of propargylic alcohol **1a** with acetylacetone (**2a**) in the presence of AuBr_3_ (5 mol%) and AgOTf (15 mol%) in [EMIM][NTf_2_] at room temperature for 30 min. This afforded propargylic substitution product **3′aa** and hydrated propargylic substitution product **3″aa** in 10% and 83% yields, respectively (Scheme 6, eq. 1). NMR spectroscopic data supported the formation of **3″aa**, and the structure was confirmed by means of X-ray crystal-structure analysis [36] (Scheme 6, eq. 1). Furthermore, the reaction of **3′aa** with 1 eq. of water in the presence of AuBr_3_ (5 mol%) and AgOTf (15 mol%) in [EMIM][NTf_2_] at room temperature for 30 min afforded **3″aa** and furan **3aa** in 86% and 13% yields, respectively, while the reaction without water gave neither **3″aa** nor **3aa** [37] (Scheme 6, eq. 2). Finally, the reaction of **3″aa** in the presence of AuBr_3_ (5 mol%) and AgOTf (15 mol%) in [EMIM][NTf_2_] at 60 °C for 30 min furnished the corresponding furan **3aa** in good yield (Scheme 6, eq. 3). These results clearly indicate that the reaction proceeds via **3′aa** and **3″aa**. 

**Scheme 7:**
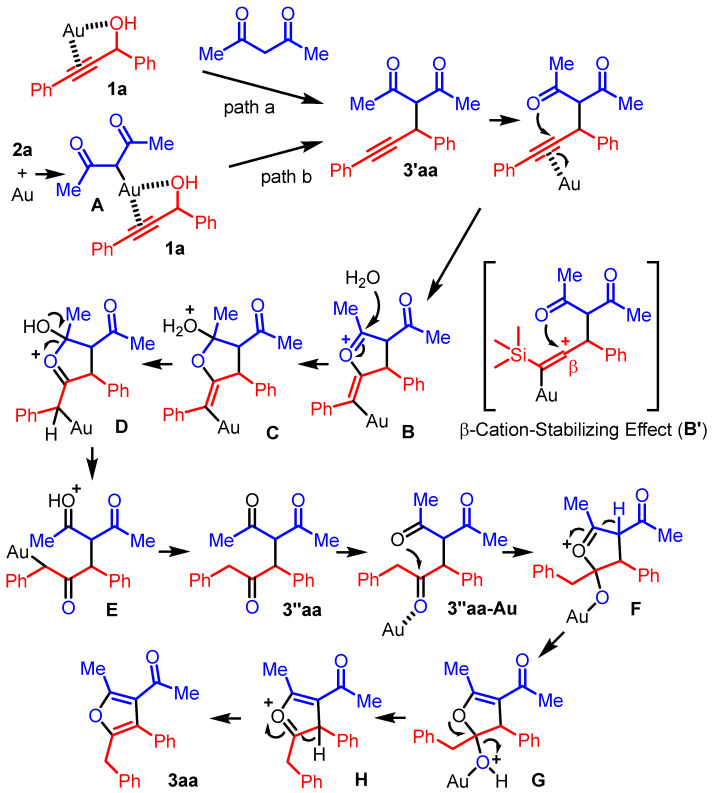
Proposed reaction mechanism of gold-catalyzed propargylic substitution followed by cycloisomerization leading to polysubstituted furan **3aa**.

A plausible reaction mechanism of gold(III)-catalyzed synthesis of polysubstituted furan **3aa** via propargylic substitution followed by cycloisomerization is shown in Scheme 7. There are two possible pathways for the formation of propargylic substitution product **3′aa** via propargylic substitution from propargylic alcohol **1a** and acetylacetone (**2a**). First, the activation of the gold catalyst by coordination to the triple bond and the oxygen atom in propargylic alcohol **1a** may promote the propargylic substitution (Scheme 7, path a). The second possibility is a propargylic substitution involving an active gold enolate species **A** [38,39,40,41,42,43,44]. That is, the reaction of gold catalyst with acetylacetone (**2a**) would produce a gold enolate species **A**, which coordinates to the triple bond and oxygen atom of the propargylic alcohol **1a**, thereby facilitating the propargylic substitution to furnish the propargylic substitution product **3′aa** (Scheme 7, path b). After completing the propargylic substitution, the gold catalyst would coordinate to the triple bond of the propargylic substitution product **3′aa**, resulting in the cyclization to afford the vinyl gold complex **B** (**3′aa** → **B**). Cyclic vinyl gold complex **B** would be opened by the addition of a water molecule to furnish the gold complex **E**, which undergoes deauration to give the dehydrated propargylic substitution product **3″aa** (**B** → **C** → **D** → **E** → **3″aa**). Finally, coordination of the gold catalyst to the carbonyl group in the intermediate **3″aa** would enable cycloisomerization, yielding the furan **3aa** (**3″aa** → **3″aa-Au** → **F** → **G** → **H** → **3aa**). This sequential reaction has been observed to be dramatically faster when the substituent on the triple bond of propargylic alcohols **1e** and **1f** is a silyl group (R = TMS) than when it is a phenyl group. This is probably due to the β-cation-stabilizing effect **B‘** [34,35] of the silyl group (TMS) on the acetylene in the cyclization from the propargylic substitution product **3′aa**. 

## 3. Materials and Methods

### 3.1. General Information

^1^H and ^13^C NMR spectra were recorded with a JEOL JNM-ECZ400S (Japan Electron Optics Laboratory Co., Ltd., Tokyo, Japan) or Bruker AV-400NEO spectrometer (Bruker, Billerica, MA, USA) at room temperature, with tetramethylsilane as an internal standard (CDCl_3_ solution). Chemical shifts were recorded in ppm, and coupling constants (*J*) in Hz. Infrared spectra (IR) were measured on an IR spectrometer (Shimadzu IRSpirit; Shimadzu Corporation, Kyoto, Japan) and are reported in wavenumbers (cm^−1^). Mass spectra were recorded on JEOL JMS-700 spectrometers (Japan Electron Optics Laboratory Co., Ltd., Tokyo, Japan). Merck silica gel 60 (1.09385) (Merck, Darmstadt, Germany) and Merck silica gel 60 F254 (Merck, Darmstadt, Germany) were used for column chromatography and thin layer chromatography (TLC), respectively. 

### 3.2. General Procedure for Gold(III)-Catalyzed Propargylic Substitution Reaction Followed by Cycloisomerization for Synthesis of Polysubstituted Furans ***3*** from Propargylic Alcohol ***1*** with 1,3-Dicarbonyl Compounds ***2***

To a solution of propargylic alcohol **1** and 1,3-dicarbonyl compound **2** in [EMIM][NTf_2_], 5 mol% of AuBr_3_ and 15 mol% of AgOTf were added at room temperature. The reaction mixture was stirred at 60 °C (propargylic alcohols **1a** for 30 min; propargylic alcohols **1e** and **1f** for 10 min). After addition of diethyl ether (5 mL) and extraction with diethyl ether (5 mL × 5), the combined organic layers were washed once with brine and dried with Na_2_SO_4_. The solvent was removed in vacuo. The residue was purified by column chromatography on silica gel with hexane and AcOEt as eluents to afford polysubstituted furan **3**.

*1-(5-Benzyl-2-methyl-4-phenylfuran-3-yl)ethan-1-one* (**3aa**): colorless oil, yield = 84%, 32 mg (hexane:AcOEt = 8:1), ^1^H-NMR (400 MHz, CDCl_3_) δ 7.41–7.38 (3H, m), 7.29–7.25 (4H, m), 7.24–7.20 (1H, m), 7.14–7.12 (2H, m), 3.81 (2H, s), 2.53 (3H, s), 1.92 (3H, s).

The ^1^H-NMR is identical with reported values [14]. 

*1-(5-Benzyl-2-ethyl-4-phenylfuran-3-yl)propan-1-one* (**3ab**): colorless oil, yield = 81%, 35 mg (hexane:AcOEt = 8:1), ^1^H-NMR (400 MHz, CDCl_3_) δ 7.40–7.37 (3H, m), 7.29–7.25 (4H, m), 7.23–7.20 (1H, m), 7.13–7.11 (2H, m), 3.82 (2H, s), 2.92 (2H, q, *J* = 7.6 Hz), 2.17 (2H, q, *J* = 7.2 Hz), 1.25 (3H, t, *J* = 7.6 Hz), 0.90 (3H, t, *J* = 7.2 Hz).

The ^1^H-NMR is identical with reported values [14]. 

*Ethyl 5-benzyl-2-methyl-4-phenylfuran-3-carboxylate* (**3ac**): colorless oil, yield = 48%, 20 mg (hexane:AcOEt = 8:1), ^1^H-NMR (400 MHz, CDCl_3_) δ 7.35–7.25 (7H, m), 7.22–7.19 (1H, m), 7.15–7.14 (2H, m), 4.10 (2H, q, *J* = 7.2 Hz), 3.85 (2H, s), 2.56 (3H, s), 1.07 (3H, t, *J* = 7.2 Hz).

The ^1^H-NMR is identical with reported values [14].

*Methyl 5-benzyl-2-methyl-4-phenylfuran-3-carboxylate* (**3ad**): colorless oil, yield = 48%, 17 mg (hexane:AcOEt = 8:1), IR (ATR) 3050, 2990, 1712, 1601 cm^−1^; ^1^H-NMR (400 MHz, CDCl_3_) δ 7.37–7.25 (7H, m), 7.23–7.20 (1H, m), 7.15–7.14 (2H, m), 3.85 (2H, s), 3.64 (3H, s), 2.56 (3H, s); ^13^C-NMR (100 MHz, CDCl_3_) δ 164.6, 158.4, 149.0, 138.2, 132.8, 129.9, 128.5, 128.3, 127.8, 127.1, 126.4, 122.6, 113.4, 51.0, 32.0, 14.3; HRMS (EI) *m*/*z* calcd for C_20_H_18_O_3_ 306.1256, found 306.1243.

*Ethyl 5-benzyl-2,4-diphenylfuran-3-carboxylate* (**3ae**): colorless oil, yield = 39%, 23 mg (hexane:AcOEt = 8:1), ^1^H-NMR (400 MHz, CDCl_3_) δ 7.81 (2H, dd, *J* = 6.6, 1.5 Hz), 7.35–7.25 (7H, m), 7.24–7.21 (1H, m), 7.15–7.13 (2H, m), 4.10 (2H, q, *J* = 7.2 Hz), 3.85 (2H, s), 2.56 (3H, m), 1.07 (3H, t, *J* = 7.2 Hz).

The ^1^H-NMR is identical with reported values [14].

*1-(5-Heptyl-2-methyl-4-phenylfuran-3-yl)ethan-1-one* (**3ca**): colorless oil, yield = 35%, 20 mg (hexane:AcOEt = 8:1), ^1^H-NMR (400 MHz, CDCl_3_) δ 7.43–7.31 (3H, m), 7.26–7.22 (2H, m), 2.54 (3H, s), 2.45 (2H, t, *J* = 7.2 Hz), 1.90 (3H, s), 1.56 (2H, br t, *J* = 7.2 Hz), 1.25–1.18 (8H, m), 0.86 (3H, t, *J* = 6.9 Hz).

The ^1^H-NMR is identical with reported values [14].

*1-(2,5-Dimethyl-4-phenylfuran-3-yl)ethan-1-one* (**3ea**): colorless oil, yield = 87%, 28 mg (hexane:AcOEt = 8:1), ^1^H-NMR (400 MHz, CDCl_3_) δ 7.42–7.34 (3H, m), 7.25–7.23 (2H, m), 2.53 (3H, s), 2.16 (3H, s), 1.93 (3H, s).

The ^1^H-NMR is identical with reported values [14,45].

*1-(2-ethyl-5-menyl-4-pheylfuran-3-yl)propan-1-one* (**3eb**): colorless oil, yield = 81%, 28 mg (hexane:AcOEt = 15:1), IR (ATR) 2980, 2939, 1761, 1697, 1672 cm^−1^; ^1^H-NMR (400 MHz, CDCl_3_) δ 7.40–7.33 (3H, m), 7.25–7.22 (2H, m), 2.91 (2H, q, *J* = 7.6 Hz), 2.18 (3H, s), 2.17 (2H, q, *J* = 7.2 Hz), 1.27 (3H, t, *J* = 7.2 Hz), 0.90 (3H, t, *J* = 7.2 Hz); ^13^C-NMR (100 MHz, CDCl_3_) δ 199.6, 160.4, 146.8, 133.9, 129.7, 128.4, 127.2, 121.9, 120.4, 35.8, 21.4, 12.5, 11.6, 8.1; HRMS (EI) *m*/*z* calcd for C_16_H_18_O_2_ 242.1307, found 242.1296.

*Ethyl 2,5-dimethyl-4-phenylfuran-3-carboxylate* (**3ec**): colorless oil, yield = 76%, 26 mg (hexane:AcOEt = 8:1), IR (ATR) 2986, 2938, 1717, 1601 cm^−1^; ^1^H-NMR (400 MHz, CDCl_3_) δ 7.35–7.31 (2H, m), 7.29–7.27 (1H, m), 7.24–7.21 (2H, m), 4.09 (2H, q, *J* = 7.2 Hz), 2.55 (3H, s), 2.18 (3H, s), 1.07 (3H, t, *J* = 7.2 Hz); ^13^C-NMR (100 MHz, CDCl_3_) δ 164.3, 157.4, 147.1, 133.3, 130.0, 127.6, 126.7, 121. 3, 113.5, 59.7, 14.0, 13.9, 11.7; HRMS (EI) *m*/*z* calcd for C_15_H_16_O_3_ 244.1099, found 244.1095.

*Methyl 2,5-dimethyl-4-phenylfuran-3-carboxylate* (**3ed**): colorless oil, yield = 83%, 26 mg (hexane:AcOEt = 8:1), IR (ATR) 2922, 1713, 1602 cm^−1^; ^1^H-NMR (400 MHz, CDCl_3_) δ 7.38–7.34 (2H, m), 7.31–7.29 (1H, m), 7.26–7.23 (2H, m), 3.65 (3H, s), 2.57 (3H, s), 2.20 (3H, s); ^13^C-NMR (100 MHz, CDCl_3_) δ 164.8, 157.6, 147.2, 133.1, 129.9, 127.7, 126.8, 121.3, 113.2, 50.9, 14.1, 11.8; HRMS (EI) *m*/*z* calcd for C_14_H_14_O_3_ 230.0943, found 230.0946.

*1-[2,5-Dimethyl-4-(naphthalen-1-yl)furan-3-yl]ethan-1-one* (**3fa**): colorless oil, yield = quant., 32 mg (hexane:AcOEt = 8:1), ^1^H-NMR (400 MHz, CDCl_3_) δ 7.91–7.87 (2H, m), 7.69–7.67 (1H, m), 7.54–7.42 (3H, m), 7.38 (1H, dd, *J* = 7.2, 1.2 Hz), 2.64 (3H, s), 2.06 (3H, s), 1.60 (3H, s).

The ^1^H-NMR is identical with reported values [14]. 

*1-[2-ethyl-5-methyl-4-(naphthalen-1-yl)furan-3-yl]propan-1-one* (**3fb**): colorless oil, yield = 97%, 36 mg (hexane:AcOEt = 8:1), IR (ATR) 3010, 2976, 2937, 1671 cm^−1^; ^1^H-NMR (400 MHz, CDCl_3_) δ 7.91–7.87 (2H, m), 7.67 (1H, br t, *J* = 8.4 Hz), 7.54–7.42 (3H, m), 7.38 (1H, dd, *J* = 7.2, 1.2 Hz), 3.07 (2H, q, *J* = 7.2 Hz), 2.08 (3H, s), 2.03–1.97 (1H, m), 1.79–1.73 (1H, m), 1.36 (3H, t, *J* = 7.6 Hz), 0.68 (3H, t, *J* = 7.2 Hz); ^13^C-NMR (100 MHz, CDCl_3_) δ 199.0, 161.5, 147.7, 133.6, 132.9, 131.6, 128.3, 128.2, 128.1, 126.5, 126.0, 125.6, 125.4, 122.5, 118.0, 34.8, 21.8, 12.3, 11.7, 7.8; HRMS (EI) *m*/*z* calcd for C_20_H_20_O_2_ 292.1463, found 292.1460.

*Ethyl 2,5-dimethyl-4-(naphthalen-1-yl)furan-3-carboxylate* (**3fc**): colorless oil, yield = 83%, 33 mg (hexane:AcOEt = 8:1), ^1^H-NMR (400 MHz, CDCl_3_) δ 7.86–7.81 (2H, m), 7.65 (1H, dd, *J* = 8.0, 0.8 Hz), 7.47–7.36 (3H, m), 7.30 (1H, dd, *J* = 6.8, 1.2 Hz), 3.78 (2H, q, *J* = 7.2 Hz), 2.66 (3H, s), 2.11 (3H, s), 0.50 (3H, t, *J* = 7.2 Hz).

The ^1^H-NMR is identical with reported values [14]. 

*Methyl 2,5-dimethyl-4-(naphthalen-1-yl)furan-3-carboxylate* (**3fd**): colorless oil, yield = 74%, 24 mg (hexane:AcOEt = 8:1), IR (ATR) 3045, 2994, 2950, 2923, 1716 cm^−1^; ^1^H-NMR (400 MHz, CDCl_3_) δ 7.87–7.82 (2H, m), 7.65–7.63 (1H, m), 7.50–7.37 (3H, m), 7.32 (1H, dd, *J* = 6.8, 1.2 Hz), 3.36 (3H, s), 2.65 (3H, s), 2.07 (3H, m); ^13^C-NMR (100 MHz, CDCl_3_) δ 164.6, 157.7, 148.0, 133.4, 132.9, 131.2, 128.1, 127.7, 127.6, 125.8, 125.7, 125.5, 125.1, 119.1, 114.6, 50.8, 14.1, 11.7; HRMS (EI) *m*/*z* calcd for C_18_H_16_O_3_ 280.1100, found 280.1096.

*3-(1,3-Diphenylprop-2-yn-1-yl)pentane-2,4-dione* (**3′aa**) and *4-Acetyl-1,3-diphenylhexene-2,5-dione* (**3″aa**): Propargylic alcohol **1a** (79 mg, 0.38 mmol) and acetylacetone (**2a**) (39 mg, 0.38 mmol) were dissolved in [EMIM][NTf_2_] (1 mL). AuBr_3_ (4.2 mg, 0.0096 mmol, 5 mol%) and AgOTf (7.4 mg, 0.029 mmol, 15 mol%) were added at room temperature, and the solution was stirred at room temperature. After the reaction was completed (monitored by thin layer chromatography), the product was extracted from the reaction mixture with diethyl ether (5 mL × 5). The combined organic layers were washed once with brine and dried with Na_2_SO_4_. The solvent was removed in vacuo. The residue was purified by column chromatography on silica gel (hexane:AcOEt = 4:1) to afford propargylic substitution product **3′aa** (29 mg, 10%) as a colorless oil and hydrated propargylic substitution product **3″aa** (98 mg, 83%) as a colorless solid.

**3′aa**: ^1^H-NMR (400 MHz, CDCl_3_) δ 7.41–7.26 (10H, m), 4.67 (1H, d, *J* = 10.8 Hz), 4.22 (1H, d, *J* = 10.8 Hz), 2.39 (3H, s), 1.93 (3H, s).

The ^1^H-NMR is identical with reported values [14,46]. 

**3″aa**: Mp. 124–126 °C; IR (ATR) 3030, 29913, 1712, 1697, 1601 cm^−1^; ^1^H-NMR (400 MHz, CDCl_3_) δ 7.36–7.30 (3H, m), 7.27–7.21 (2H, m), 7.17–7.15 (2H, m), 6.97–6.95 (2H, m), 4.64 (1H, d, *J* = 11.2 Hz), 4.60 (1H, d, *J* = 11.2 Hz), 3.69 (2H, s), 2.24 (3H, s), 1.87 (3H, s); ^13^C-NMR (100 MHz, CDCl_3_) δ 205.6, 202.5, 201.5, 134.1, 133.5, 129.6, 129.4, 129.0, 128.4, 128.3, 127.0, 70.6, 57.7, 48.0, 30.9, 30.1; HRMS (EI) *m*/*z* calcd for C_20_H_20_O_3_ 308.1412, found 308.1417.

### 3.3. General Procedure for Catalyst Recycling in the Gold-Catalyzed Propargylic Substitution Followed by Cycloisomerization

AuBr_3_ (5 mol%) and AgOTf (15 mol%) were added to a solution of propargylic alcohol **1f** and acetylacetone (**2a**) in [EMIM][NTf_2_] (1 mL) at room temperature, and the mixture was stirred at 60 °C. After complete consumption of propargylic alcohol **1f**, the product was extracted from the reaction mixture with Et_2_O (5 mL × 5). The diethyl ether layer was separated. The ionic liquid layer containing the gold catalyst was under reduced pressure for 1 h to remove the ether and water contained and reused for the next reaction.

## 4. Conclusions

We present an efficient synthesis of polysubstituted furans in ionic liquid via gold-catalyzed propargylic substitution followed by cycloisomerization. This reaction provides an alternative, environmentally friendly method for the synthesis of various polysubstituted furans. We are currently applying it to synthesize biologically important compounds containing furan skeletons. Experimental and theoretical investigations of the reaction mechanism are also in progress. 

## Figures and Tables

**Table 1:** Optimization of the reaction conditions for gold-catalyzed propargylic substitution followed by cycloisomerization for the synthesis of polysubstituted furan **3aa**.

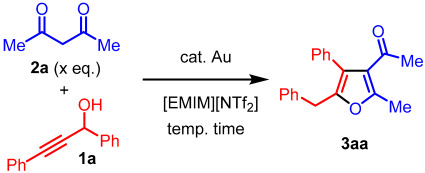					
Entry	Cat. Au (mol%)	X eq.	Temp.	Time	3aa Yield
1	AuBr_3_ (5)	1	r.t.	3 days	36%
2	AuBr_3_ (5)/AgOTf (15)	1	r.t.	2 days	72%
3	AuBr_3_ (5)/AgOTf (15)	1	100 °C	30 min	48%
4	AuBr_3_ (5)/AgOTf (15)	1	60 °C	30 min	84%
5	AuBr_3_ (5)/AgOTf (15)	3	60 °C	30 h	71%
6	AuCl (5)	1	r.t.	3 days	30%
7	AuCl (5)/AgOTf (5)	1	r.t.	3 days	46%

**Table 2:** Effect of the counter anion of the silver catalyst.

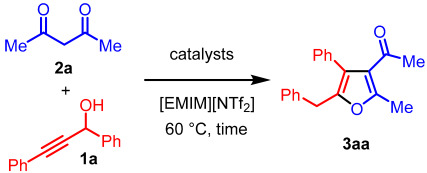			
Entry	Catalysts	Time	3aa Yield
1	AuBr_3_ (5)/AgOTf (15)	30 min	84%
2	AuBr_3_ (5)/AgNTf_2_ (15)	1 day	57%
3	AuBr_3_ (5)/AgBF_4_ (15)	2 days	47%
4	AuBr_3_ (5)/AgSbF_6_ (15)	2 days	n.d.

**Table 3:** Solvent effect in gold-catalyzed propargylic substitution followed by cycloisomerization.

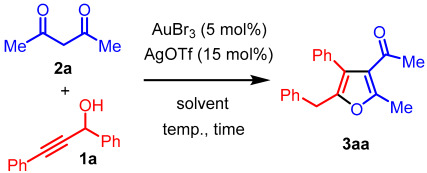			
Entry	Solvent	Temp. (Time)	3aa Yield
1	[EMIM][NTf_2_]	60 °C (30 min)	84%
2	[EMIM][CH_3_CO_2_]	r.t. (1 h) to 100 °C (1 day)	no reaction
3	[EMIM][Me_2_PO_4_]	r.t. (1 h) to 100 °C (1 day)	no reaction
4	[EMIM][HSO_4_]	r.t. (1 h) to 100 °C (1 day)	trace
5	[EMIM][MeSO_4_]	r.t. (1 h) to 100 °C (1 day)	trace
6	[EMIM][OTf]	r.t. (1 h) to 100 °C (1 day)	trace
7	ClCH_2_CH_2_Cl	60 °C (3 h)	49%

**Table 4:** Catalyst recycling in the gold-catalyzed propargylic substitution of propargylic alcohol **1f** with acetylacetone (**2a**) followed by cycloisomerization.

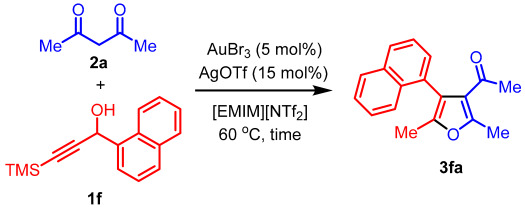		
Run	Time	3fa Yield
1	10 min	Quant.
2	3 h	90%
3	17 h	89%

## Data Availability

Data are contained within the article and Supplementary Materials.

## Supplementary Materials

The following supporting information can be downloaded at: https://www.mdpi.com/article/10.3390/molecules29225441/s1, ^1^H, ^13^C-NMR spectrum.
