# Supplementary material for: Gold-Catalyzed Propargylic Substitution Followed by Cycloisomerization in Ionic Liquid: Environmentally Friendly Synthesis of Polysubstituted Furans from Propargylic Alcohols and 1,3-Dicarbonyl Compounds
Source: Molecules. 2024 Nov 18;29(22):5441. doi: 10.3390/molecules29225441 (PMC11597079; doi:10.3390/molecules29225441)
Supplement: Supplementary file 1 [file molecules-29-05441-s001.zip › molecules-3265961-supplementary.pdf]

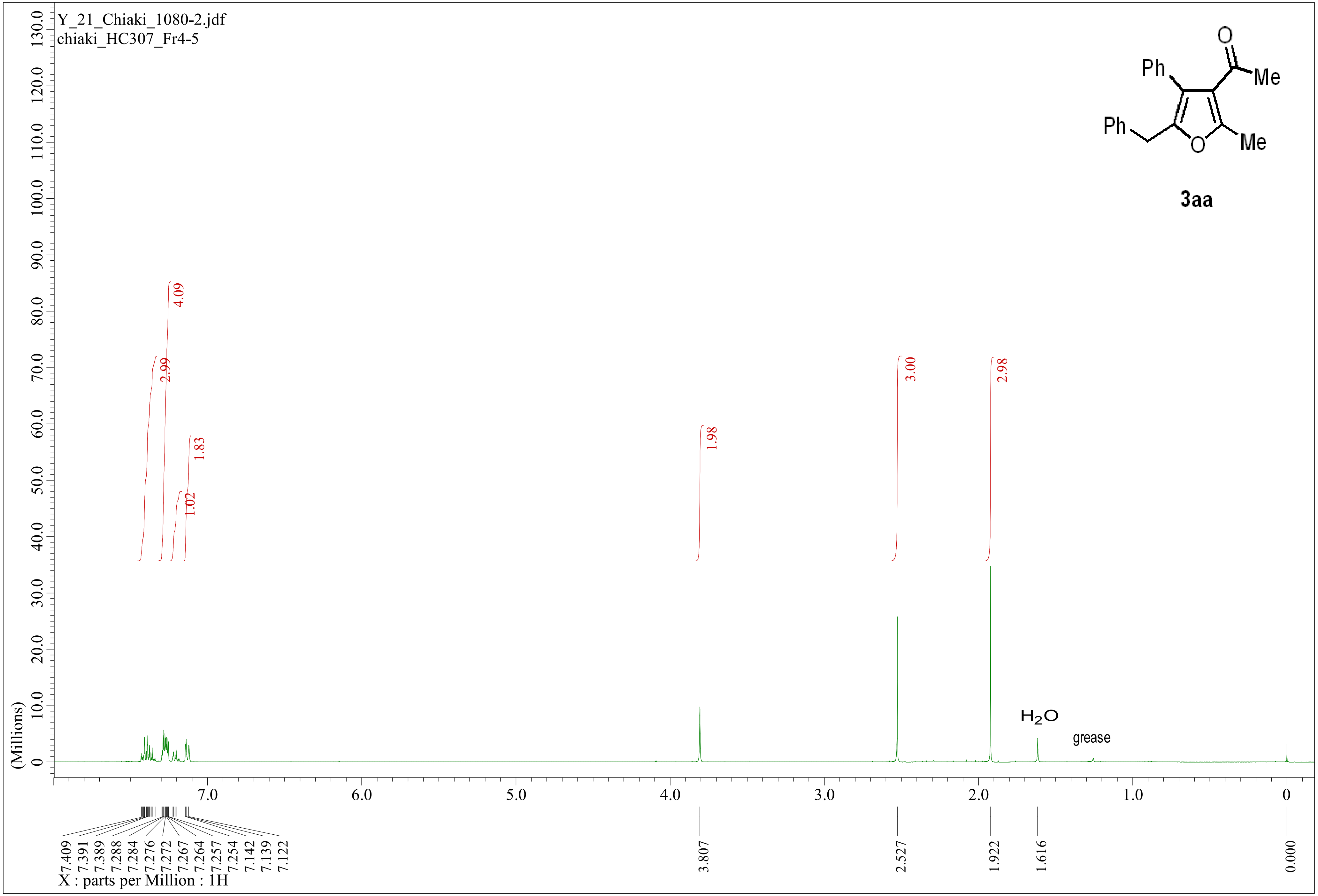
Y_21_Chiaki_1080-2.jdf
chiaki_HC307_Fr4-5
Ph
O
Me
Ph
O
Me
3aa
(Millions)
130.0
120.0
110.0
100.0
90.0
80.0
70.0
60.0
50.0
40.0
30.0
20.0
10.0
0
4.09
2.99
1.83
1.02
1.98
3.00
2.98
H2O
grease
7.0
6.0
5.0
4.0
3.0
2.0
1.0
0
7.409
7.391
7.389
7.288
7.284
7.276
7.272
7.267
7.264
7.257
7.254
7.142
7.139
7.122
3.807
2.527
1.922
1.616
0.000
X : parts per Million : 1H

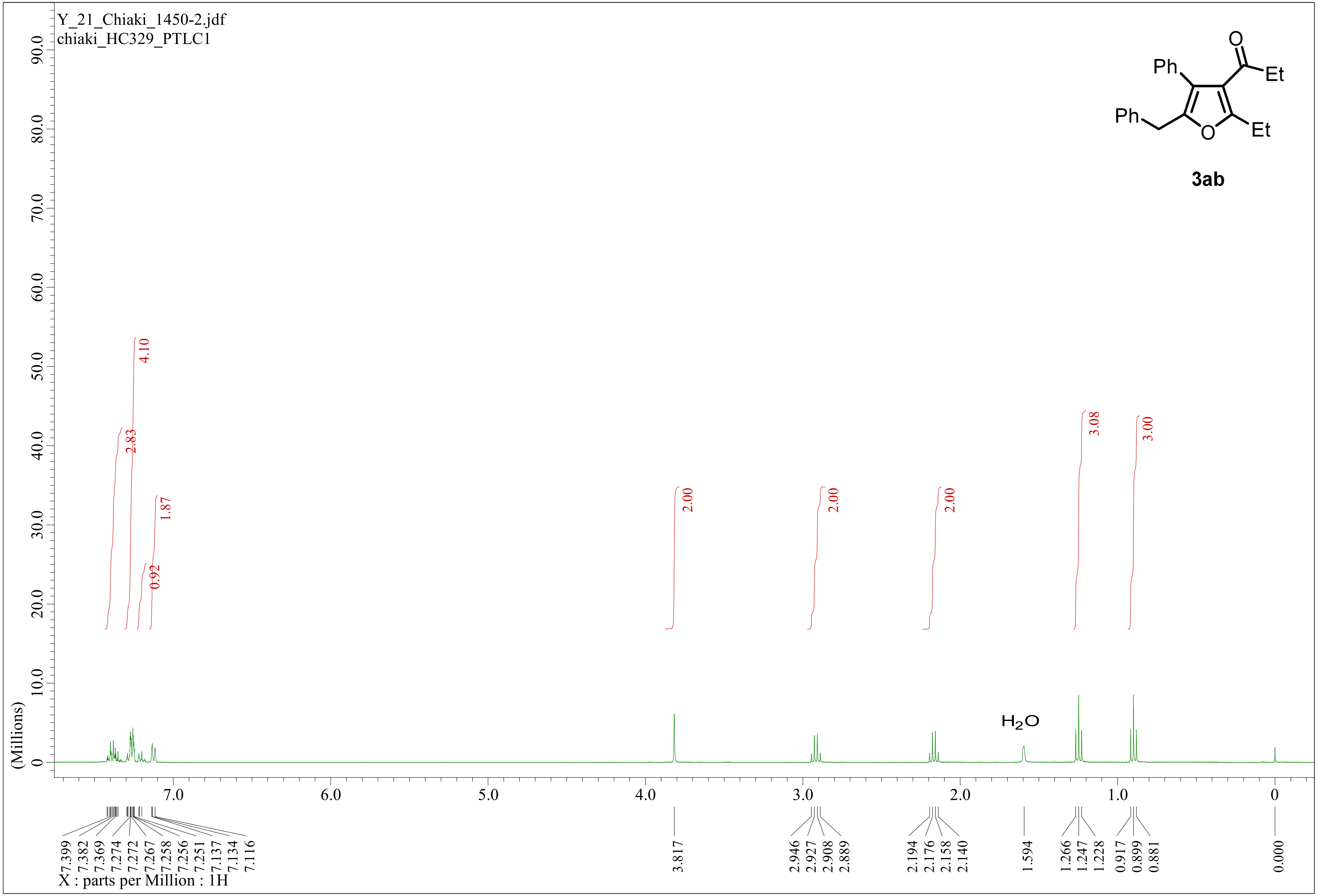
Y_21_Chiaki_1450-2.jdf
chiaki_HC329_PTLC1
Ph
O
Et
Ph
O
Et
3ab
H2O
(Millions)
0
10.0
20.0
30.0
40.0
50.0
60.0
70.0
80.0
90.0
4.10
2.83
0.92
1.87
2.00
2.00
2.00
3.08
3.00
7.0
6.0
5.0
4.0
3.0
2.0
1.0
0
7.399
7.382
7.369
7.274
7.272
7.267
7.258
7.256
7.251
7.137
7.134
7.116
3.817
2.946
2.927
2.908
2.889
2.194
2.176
2.158
2.140
1.594
1.266
1.247
1.228
0.917
0.899
0.881
0.000
X : parts per Million : 1H

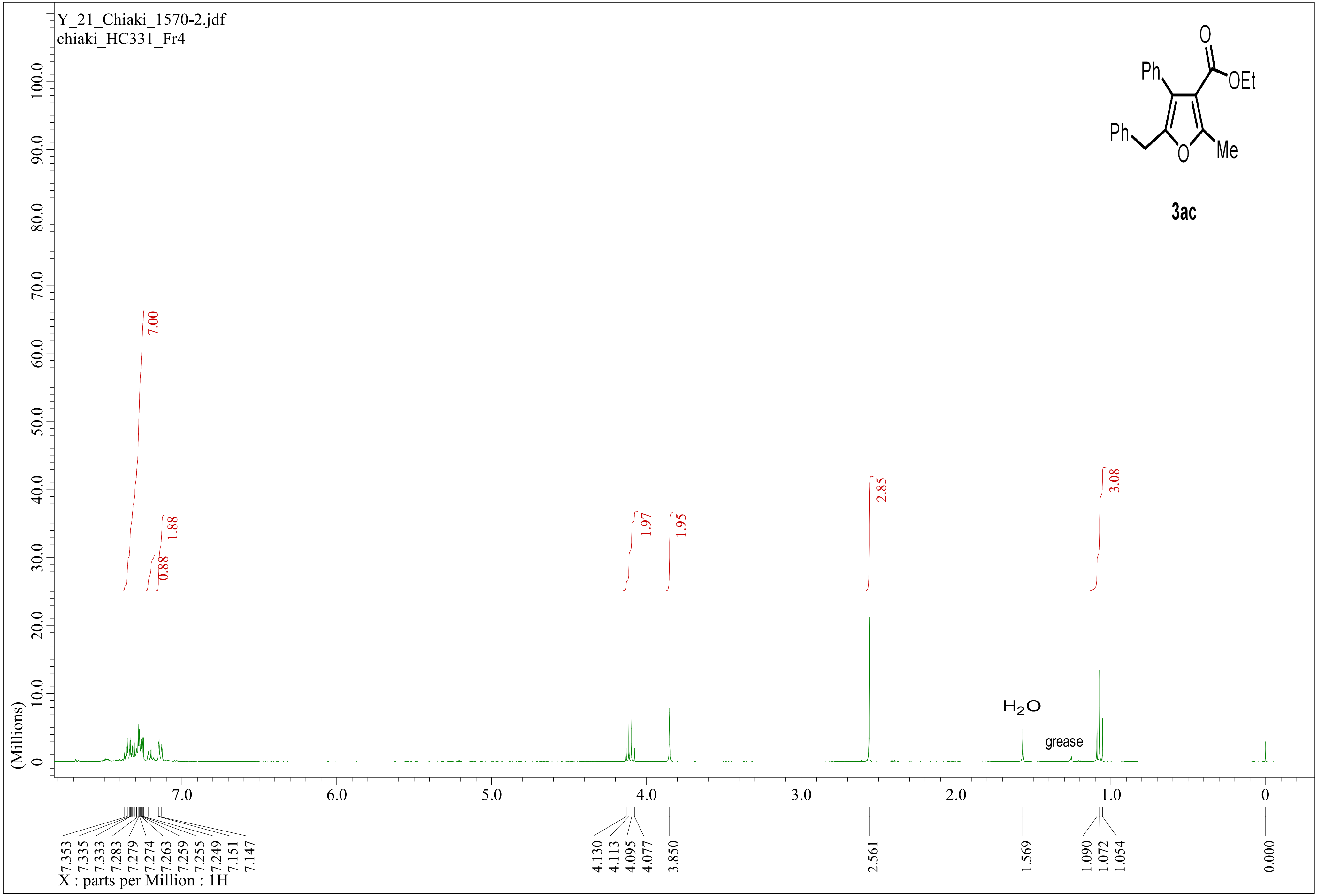
Y_21_Chiaki_1570-2.jdf
chiaki_HC331_Fr4
Ph
O
OEt
Ph
O
Me
3ac
7.00
1.88
0.88
1.97
1.95
2.85
3.08
H2O
grease
(Millions)
7.353
7.335
7.333
7.283
7.279
7.274
7.263
7.259
7.255
7.249
7.151
7.147
4.130
4.113
4.095
4.077
3.850
2.561
1.569
1.090
1.072
1.054
0.000
X : parts per Million : 1H

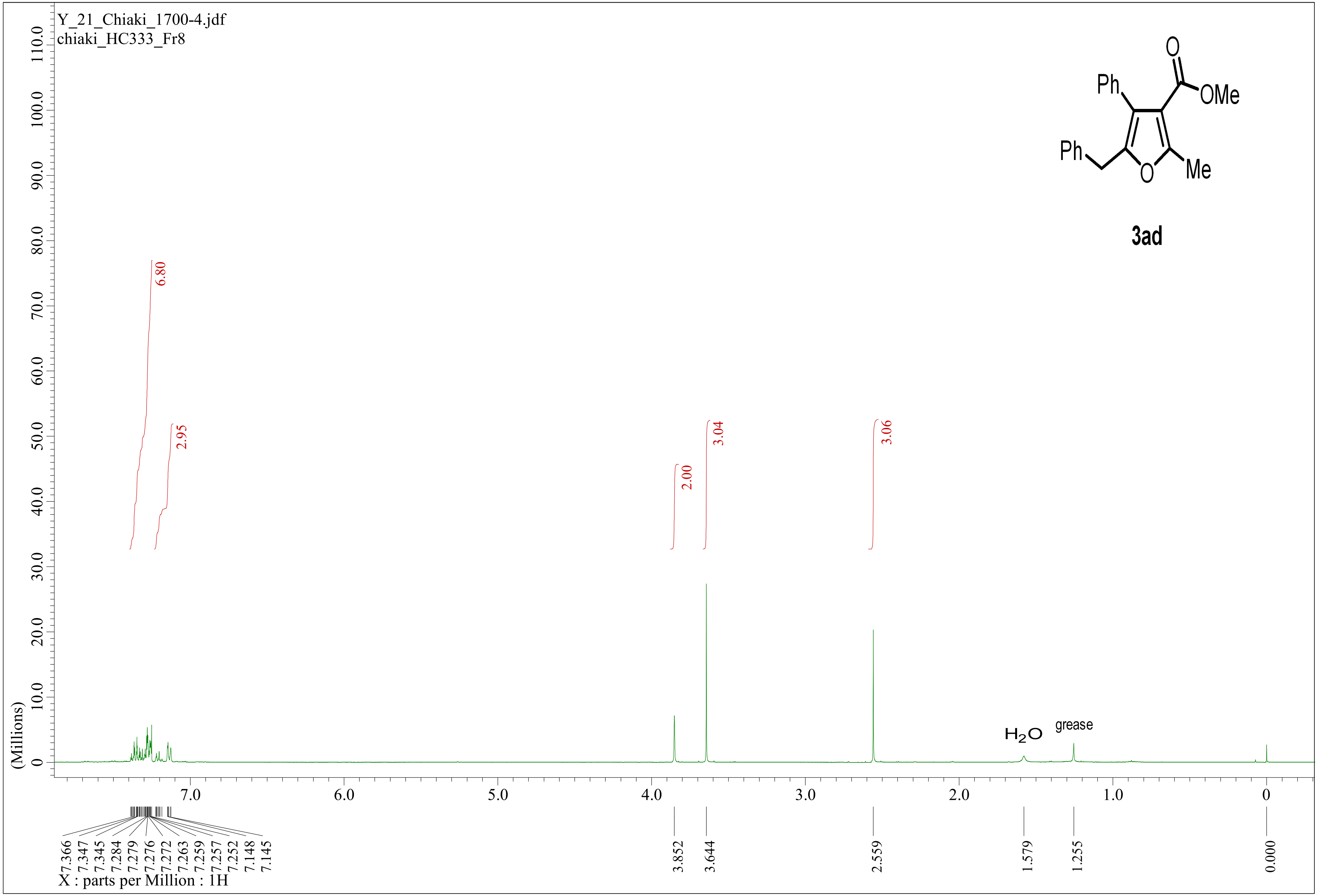
Y_21_Chiaki_1700-4.jdf
chiaki_HC333_Fr8
Ph
O
OMe
Ph
O
Me
3ad
6.80
2.95
2.00
3.04
3.06
H2O
grease
(Millions)
0
10.0
20.0
30.0
40.0
50.0
60.0
70.0
80.0
90.0
100.0
110.0
7.0
6.0
5.0
4.0
3.0
2.0
1.0
0
7.366
7.347
7.345
7.284
7.279
7.276
7.272
7.263
7.259
7.257
7.252
7.148
7.145
3.852
3.644
2.559
1.579
1.255
0.000
X : parts per Million : 1H

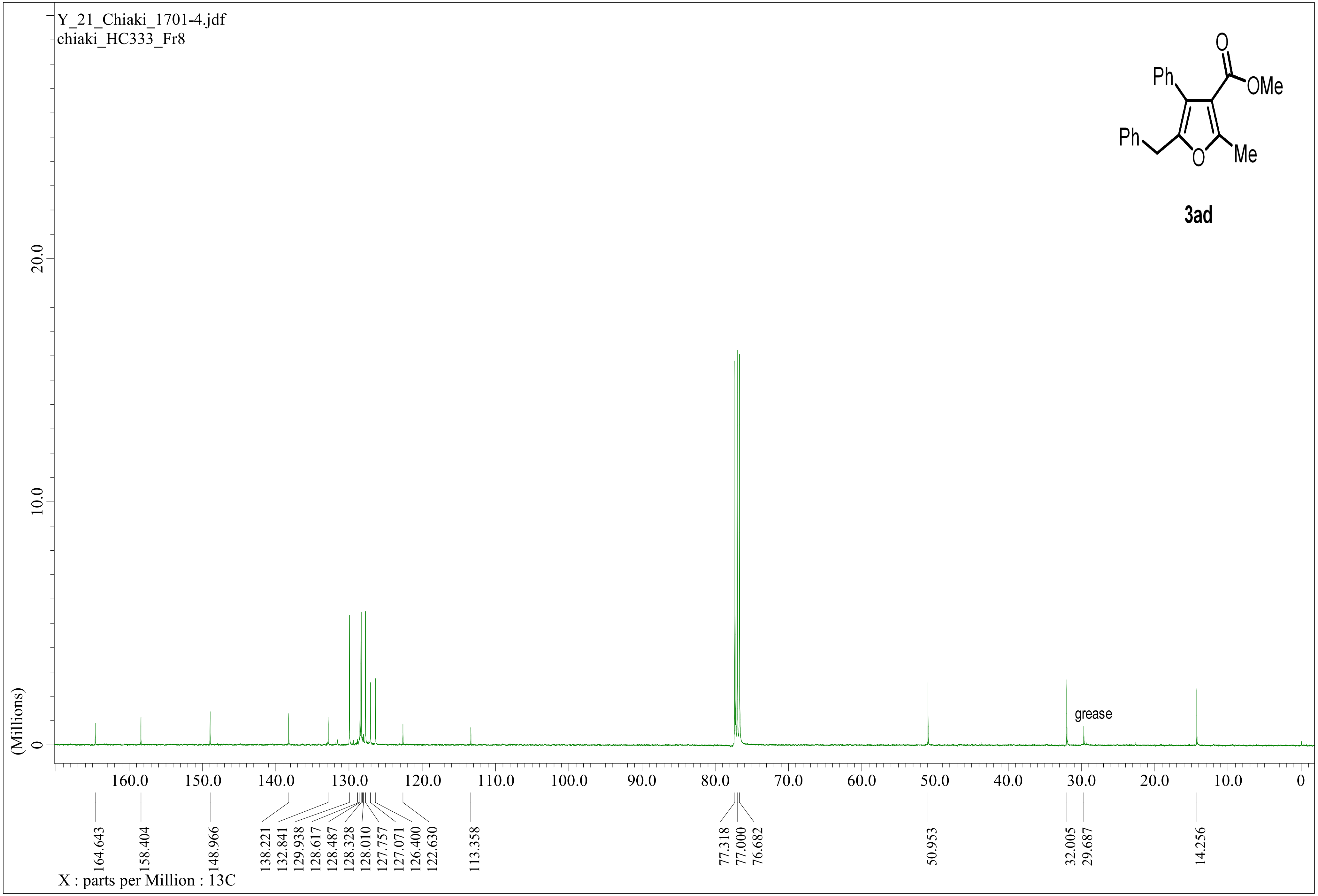
Y_21_Chiaki_1701-4.jdf
chiaki_HC333_Fr8
Ph
O
OMe
Ph
O
Me
3ad
grease
(Millions)
0
10.0
20.0
160.0
150.0
140.0
130.0
120.0
110.0
100.0
90.0
80.0
70.0
60.0
50.0
40.0
30.0
20.0
10.0
0
164.643
158.404
148.966
138.221
132.841
129.938
128.617
128.487
128.328
128.010
127.757
127.071
126.400
122.630
113.358
77.318
77.000
76.682
50.953
32.005
29.687
14.256
X : parts per Million : 13C

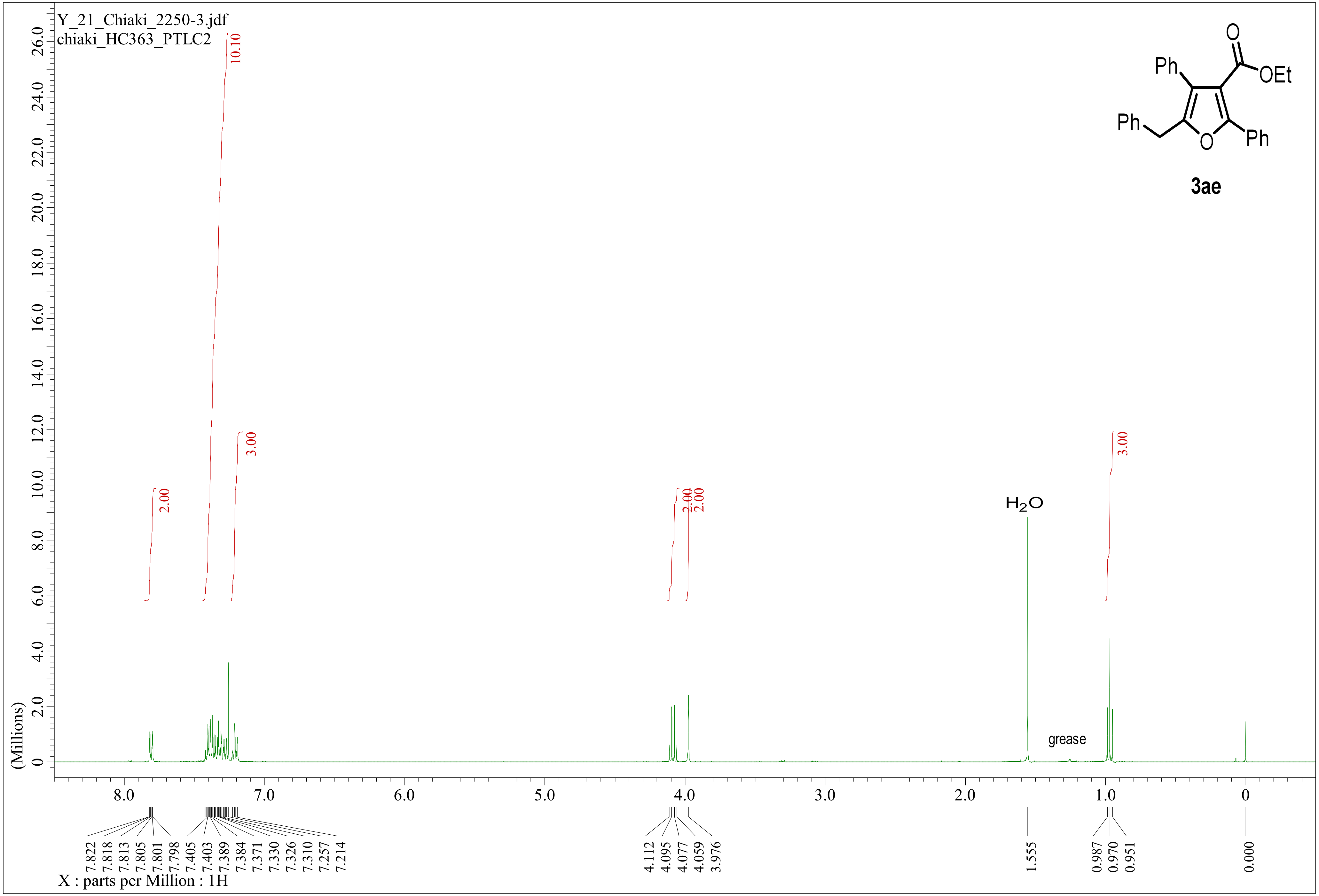
Y_21_Chiaki_2250-3.jdf
chiaki_HC363_PTLC2
3ae
Ph
Ph
Ph
O
O
OEt
H2O
grease
2.00
10.10
3.00
2.00
2.00
3.00
(Millions)
0
2.0
4.0
6.0
8.0
10.0
12.0
14.0
16.0
18.0
20.0
22.0
24.0
26.0
8.0
7.0
6.0
5.0
4.0
3.0
2.0
1.0
0
7.822
7.818
7.813
7.805
7.801
7.798
7.405
7.403
7.389
7.384
7.371
7.330
7.326
7.310
7.257
7.214
4.112
4.095
4.077
4.059
3.976
1.555
0.987
0.970
0.951
0.000
X : parts per Million : 1H

161201NM518

¥¥192.168.226.211¥anal¥森田¥161201NM5181NON_E59.als

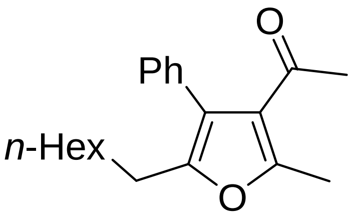

DFILE 161201NM5181NON_E59.als
COMNT 161201NM518
DATIM Thu Dec 01 16:05:26 2016
OBNUC 1H
EXMOD NON
OBFRQ 300.40 MHz
OBSET 130.00 KHz
OBFIN 1150.00 Hz
POINT 16384
FREQU 6006.01 Hz
SCANS 8
ACQTM 2.7279 sec
PD 4.2720 sec
PW1 6.00 usec
IRNUC 1H
CTEMP 21.6 c
SLVNT CDCL3
EXREF 0.00 ppm
BF 0.12 Hz
RGAIN 15

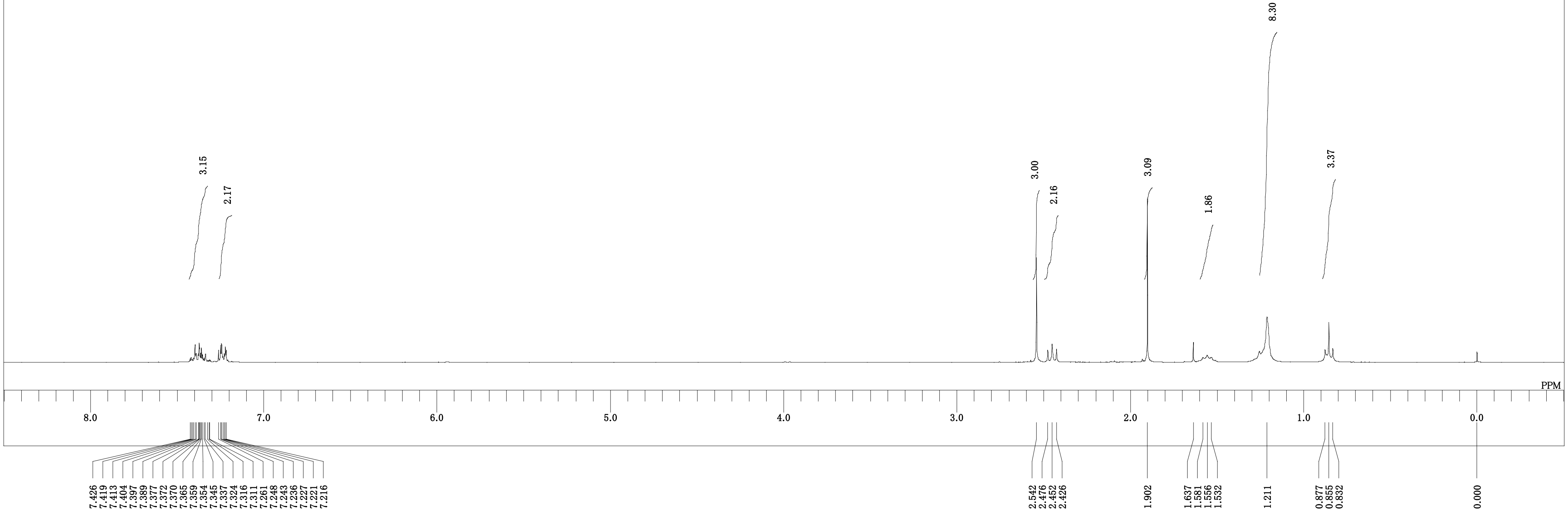

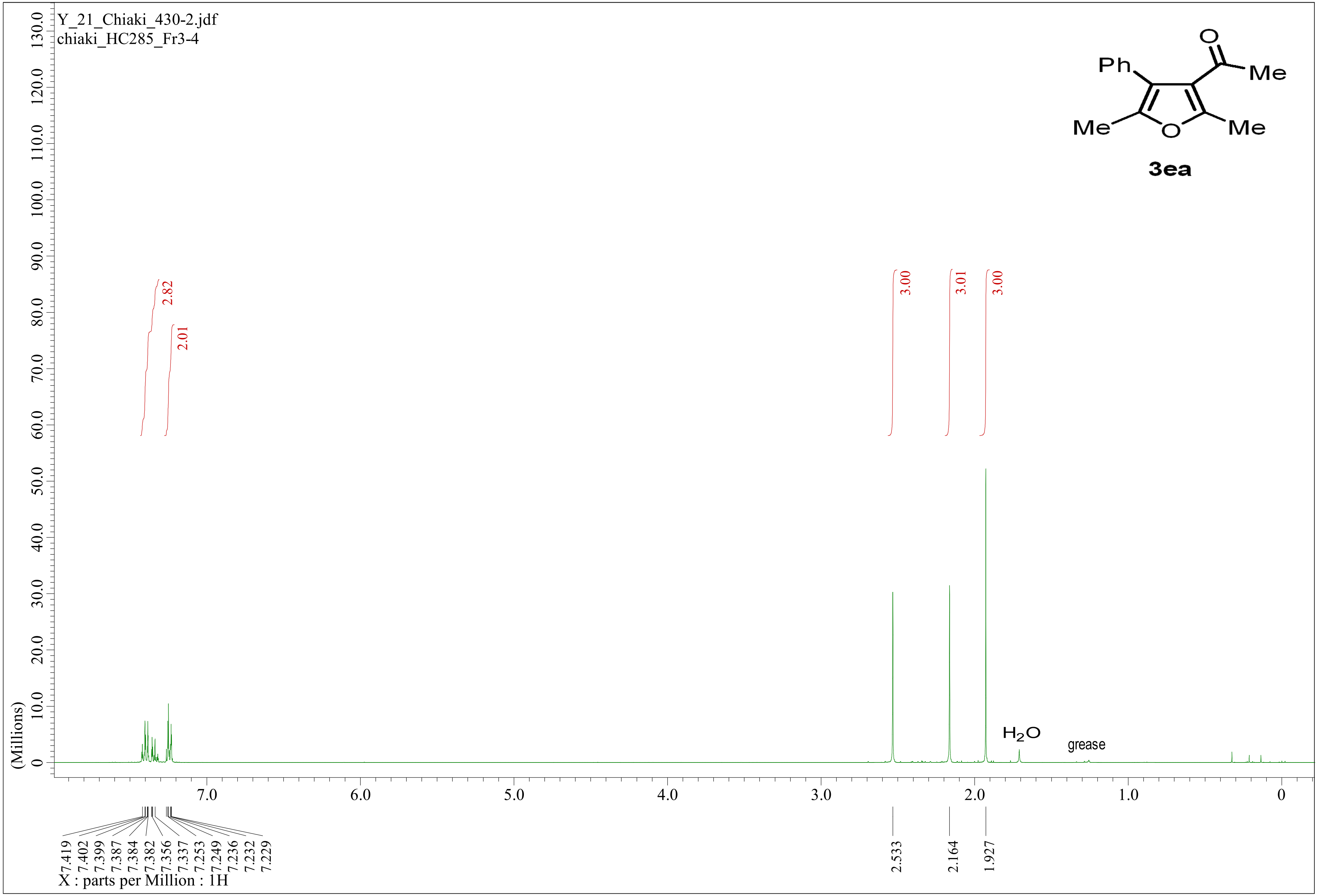
Y_21_Chiaki_430-2.jdf
chiaki_HC285_Fr3-4
Ph
Me
Me
O
Me
O
3ea
2.82
2.01
3.00
3.01
3.00
H2O
grease
(Millions)
X : parts per Million : 1H
7.419
7.402
7.399
7.387
7.384
7.382
7.356
7.337
7.253
7.249
7.236
7.232
7.229
2.533
2.164
1.927

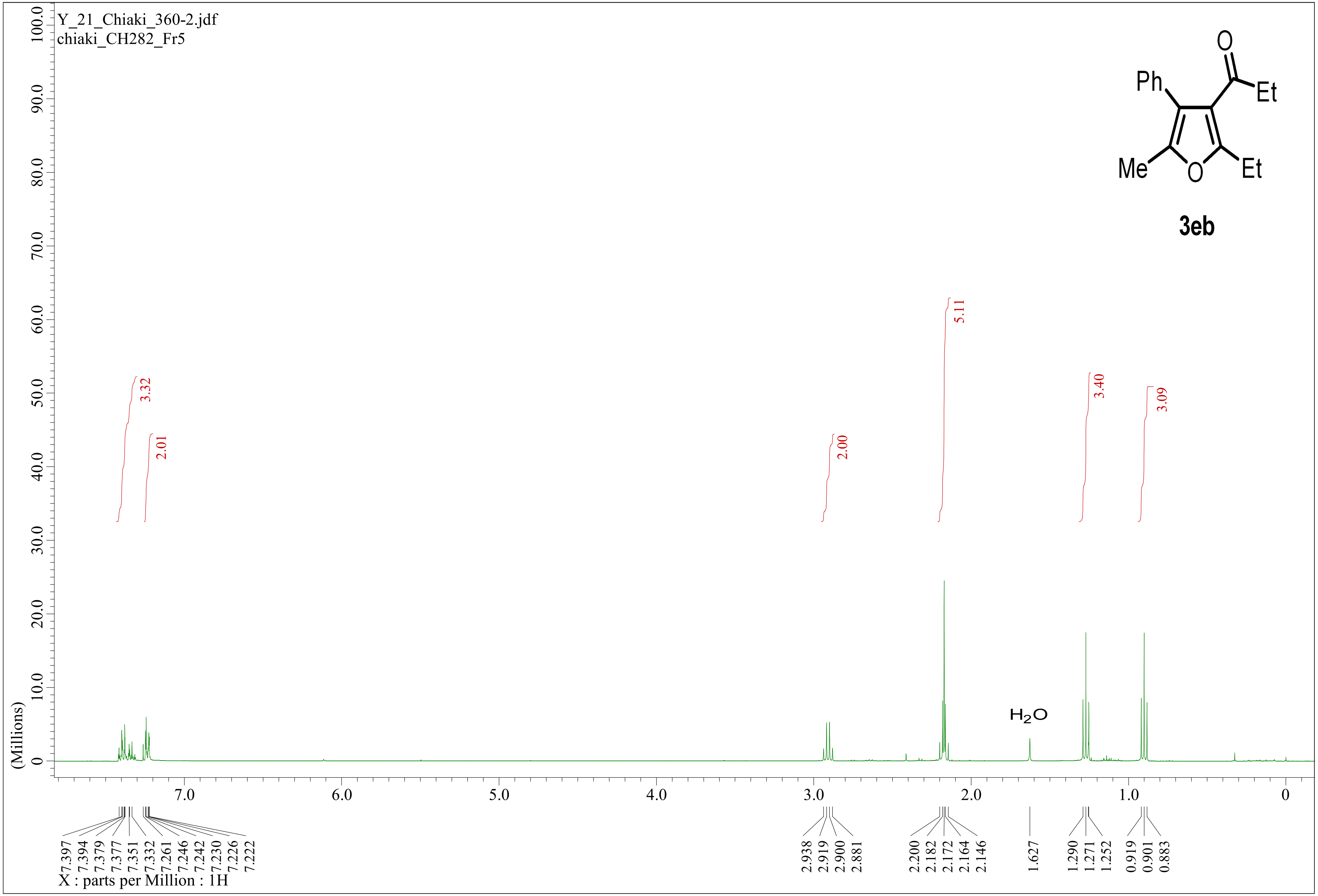
Y_21_Chiaki_360-2.jdf
chiaki_CH282_Fr5
Ph
O
Et
Me
O
Et
3eb
3.32
2.01
2.00
5.11
3.40
3.09
H2O
(Millions)
X : parts per Million : 1H
7.397 7.394 7.379 7.377 7.351 7.332 7.261 7.246 7.242 7.230 7.226 7.222
2.938 2.919 2.900 2.881
2.200 2.182 2.172 2.164 2.146
1.627
1.290 1.271 1.252
0.919 0.901 0.883

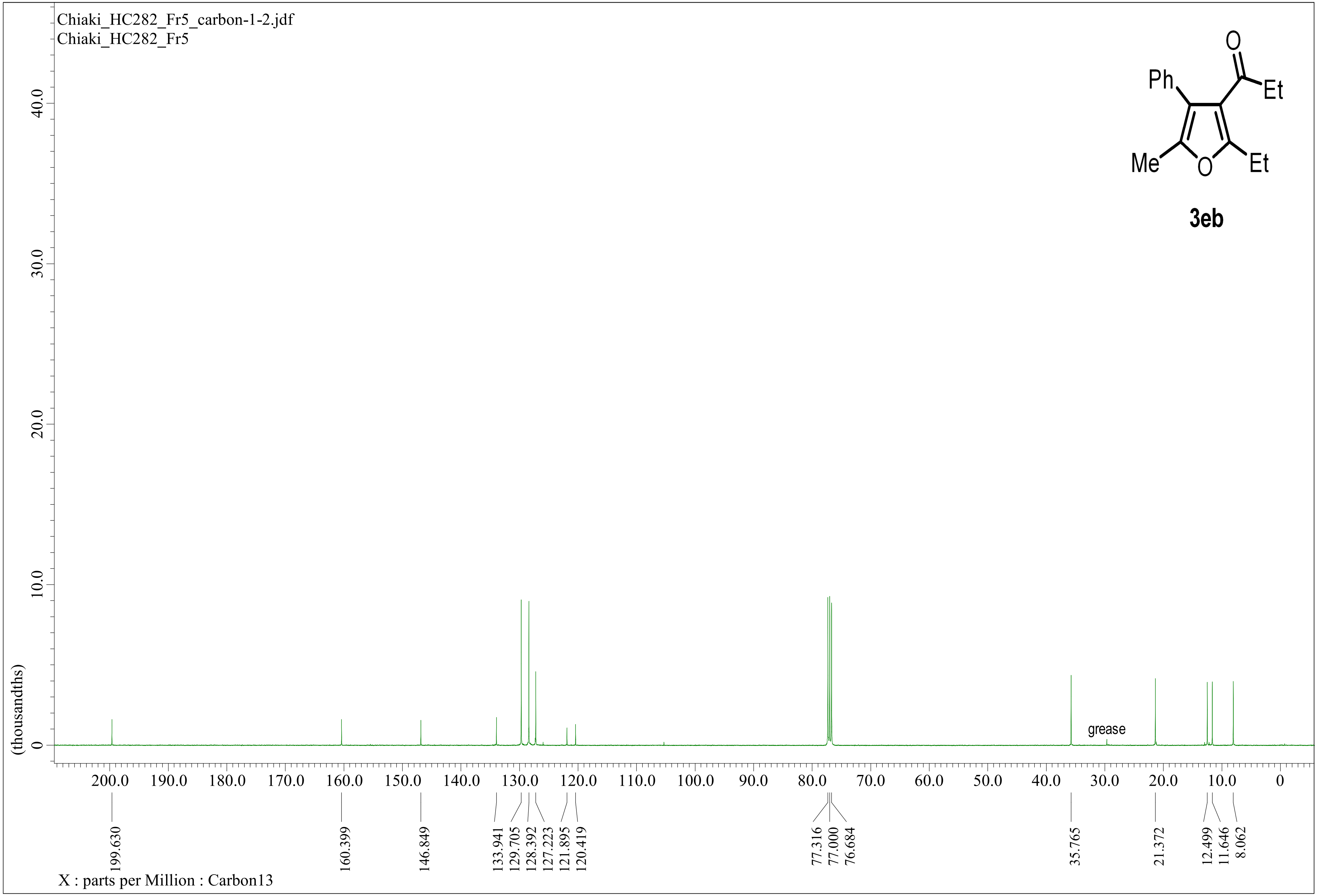
Chiaki_HC282_Fr5_carbon-1-2.jdf
Chiaki_HC282_Fr5
(thousandths)
40.0
30.0
20.0
10.0
0
grease
200.0 190.0 180.0 170.0 160.0 150.0 140.0 130.0 120.0 110.0 100.0 90.0 80.0 70.0 60.0 50.0 40.0 30.0 20.0 10.0 0
199.630
160.399
146.849
133.941
129.705
128.392
127.223
121.895
120.419
77.316
77.000
76.684
35.765
21.372
12.499
11.646
8.062
X : parts per Million : Carbon13
O
Ph
Et
Me
O
Et

3eb

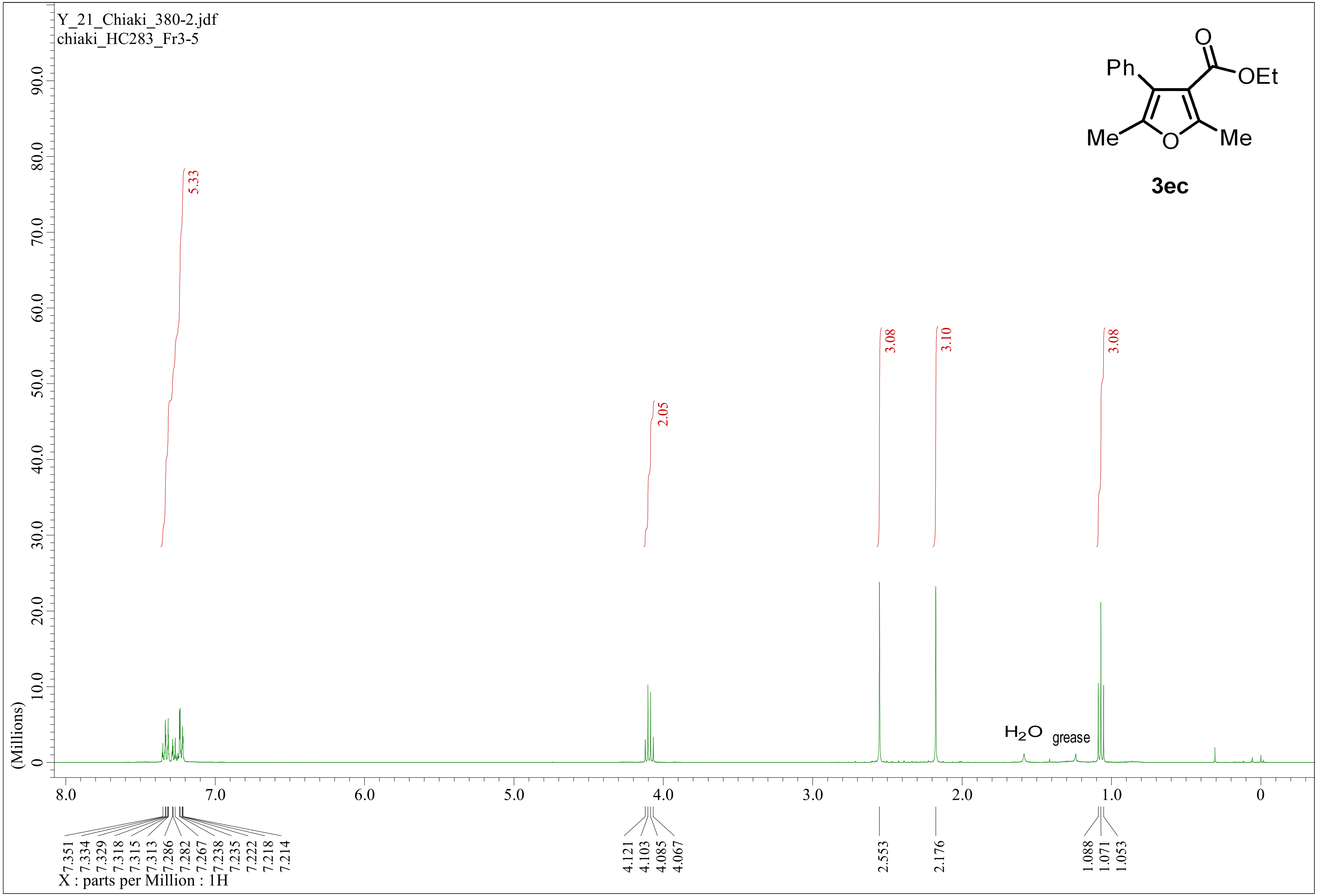

Y_21_Chiaki_380-2.jdf
chiaki_HC283_Fr3-5
Ph
O
OEt
Me
O
Me
3ec
5.33
2.05
3.08
3.10
3.08
H_2O
grease
(Millions)
0
10.0
20.0
30.0
40.0
50.0
60.0
70.0
80.0
90.0
8.0
7.0
6.0
5.0
4.0
3.0
2.0
1.0
0
7.351
7.334
7.329
7.318
7.315
7.313
7.286
7.282
7.267
7.238
7.235
7.222
7.218
7.214
4.121
4.103
4.085
4.067
2.553
2.176
1.088
1.071
1.053
X : parts per Million : 1H

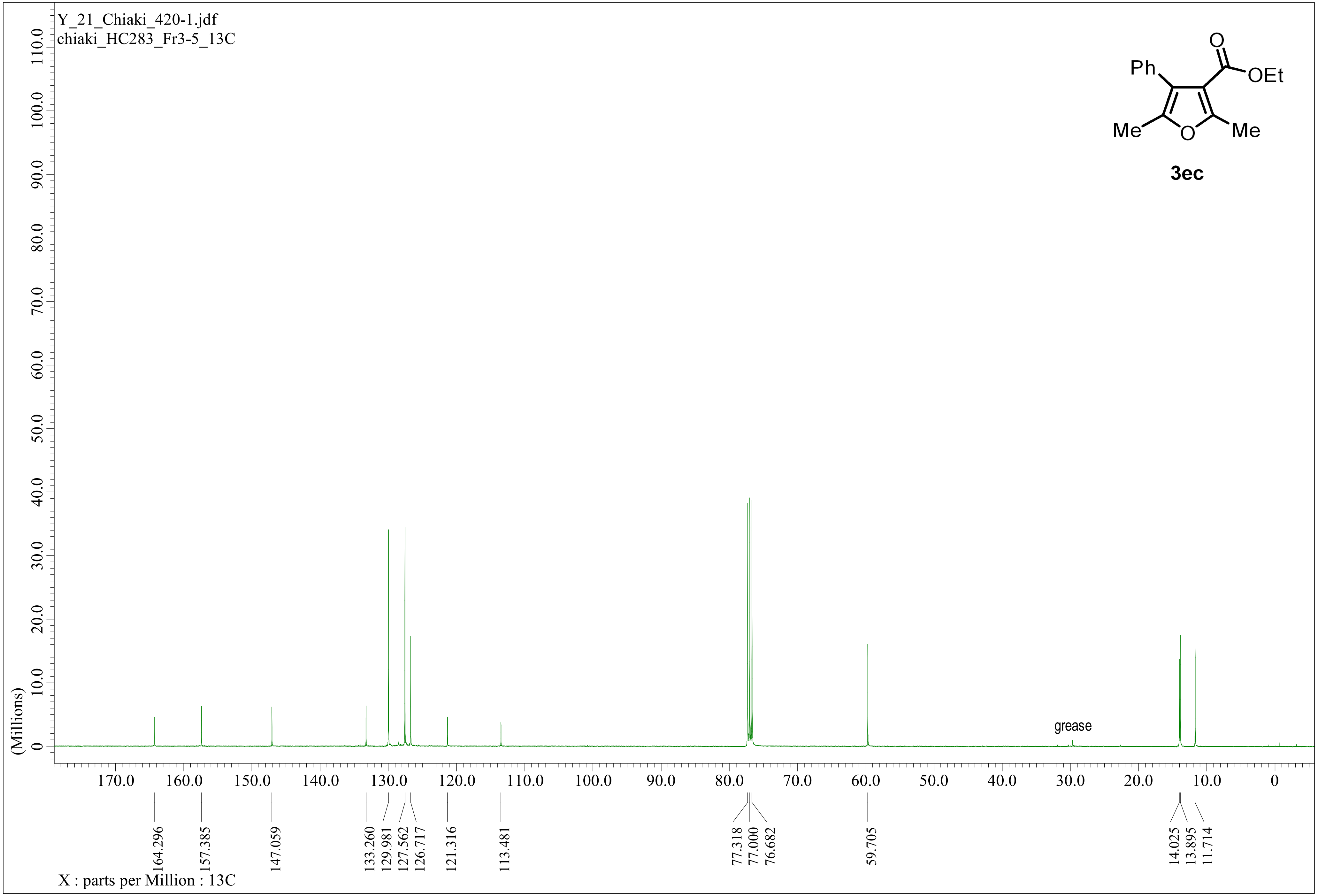
Y_21_Chiaki_420-1.jdf
chiaki_HC283_Fr3-5_13C
Ph
O
OEt
Me
O
Me
3ec
(Millions)
0
10.0
20.0
30.0
40.0
50.0
60.0
70.0
80.0
90.0
100.0
110.0
grease
170.0
160.0
150.0
140.0
130.0
120.0
110.0
100.0
90.0
80.0
70.0
60.0
50.0
40.0
30.0
20.0
10.0
0
164.296
157.385
147.059
133.260
129.981
127.562
126.717
121.316
113.481
77.318
77.000
76.682
59.705
14.025
13.895
11.714
X : parts per Million : 13C

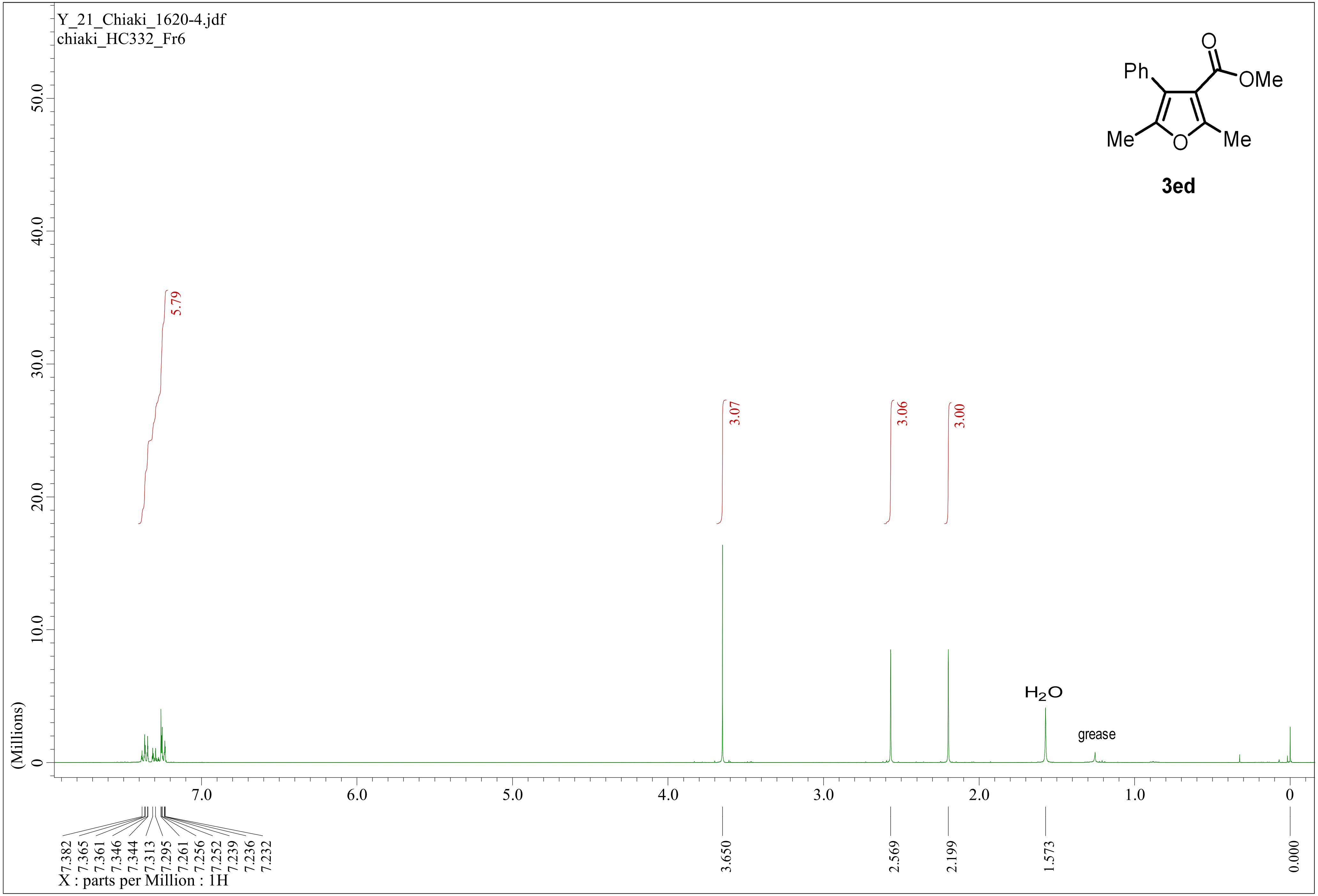
Y_21_Chiaki_1620-4.jdf
chiaki_HC332_Fr6
Ph
O
OMe
Me
O
Me
3ed
5.79
3.07
3.06
3.00
H_2O
grease
(Millions)
50.0
40.0
30.0
20.0
10.0
0
7.0
6.0
5.0
4.0
3.0
2.0
1.0
0
7.382
7.365
7.361
7.346
7.344
7.313
7.295
7.261
7.256
7.252
7.239
7.236
7.232
3.650
2.569
2.199
1.573
0.000
X : parts per Million : 1H

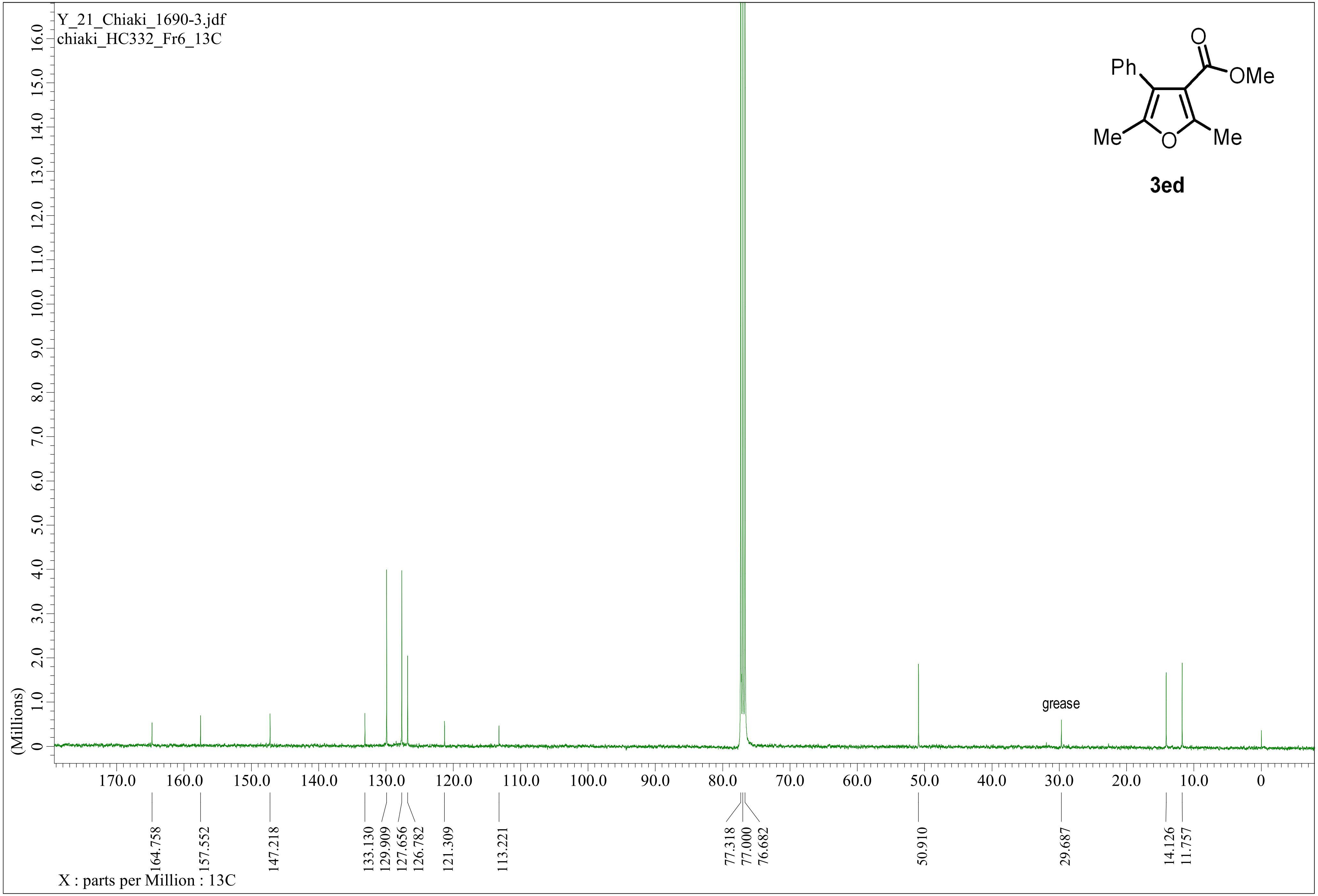
Y_21_Chiaki_1690-3.jdf
chiaki_HC332_Fr6_13C
Ph
O
OMe
Me
O
Me
3ed
(Millions)
grease
164.758
157.552
147.218
133.130
129.909
127.656
126.782
121.309
113.221
77.318
77.000
76.682
50.910
29.687
14.126
11.757
X : parts per Million : 13C

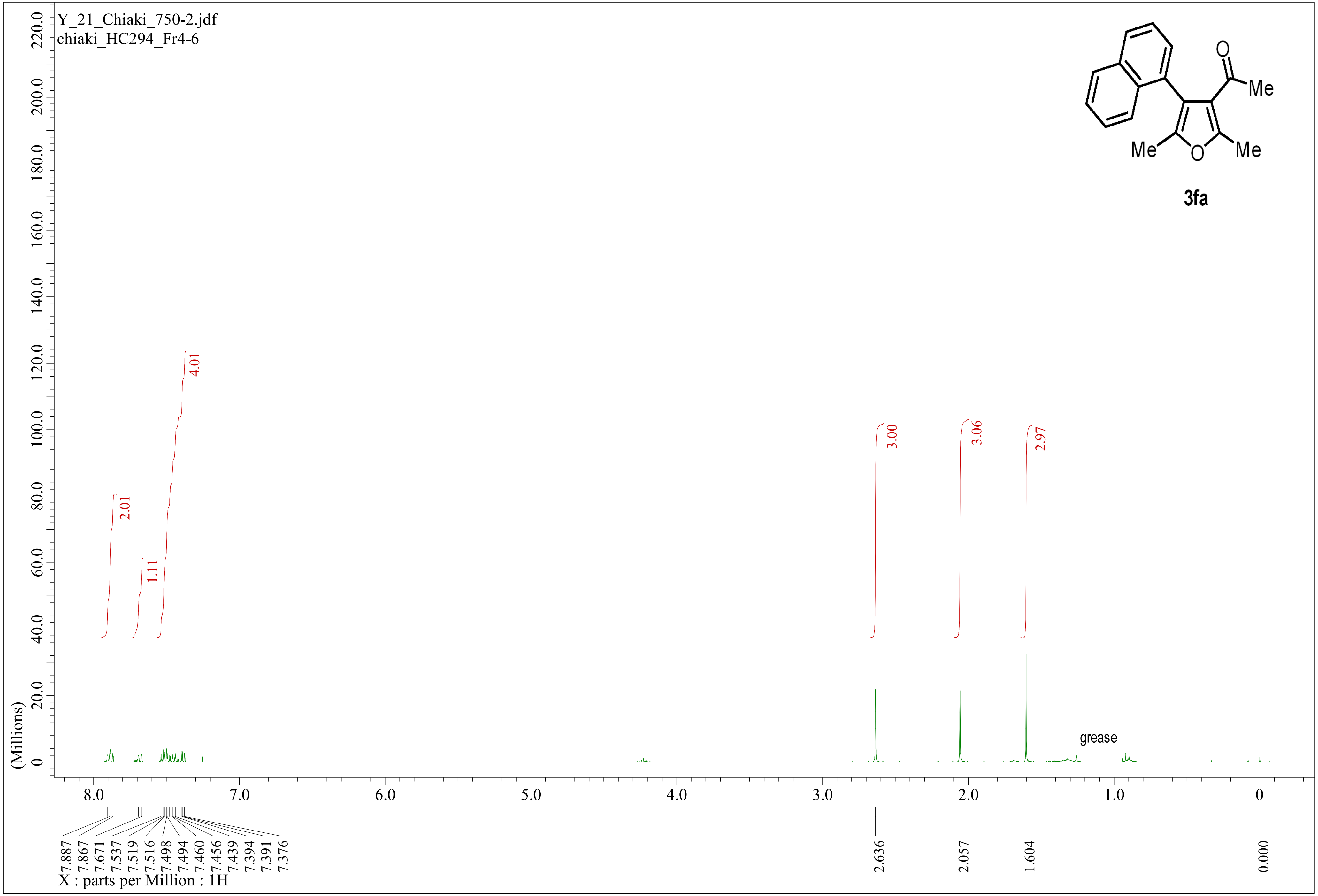
Y_21_Chiaki_750-2.jdf
chiaki_HC294_Fr4-6
O
Me
Me
O
Me
3fa
grease
2.01
1.11
4.01
3.00
3.06
2.97
7.887
7.867
7.671
7.537
7.519
7.516
7.498
7.494
7.460
7.456
7.439
7.394
7.391
7.376
2.636
2.057
1.604
0.000
8.0 7.0 6.0 5.0 4.0 3.0 2.0 1.0 0
X : parts per Million : 1H
(Millions)
0 20.0 40.0 60.0 80.0 100.0 120.0 140.0 160.0 180.0 200.0 220.0

Y_21_Chiaki_1780-3.jdf
chiaki_HC335_Fr5

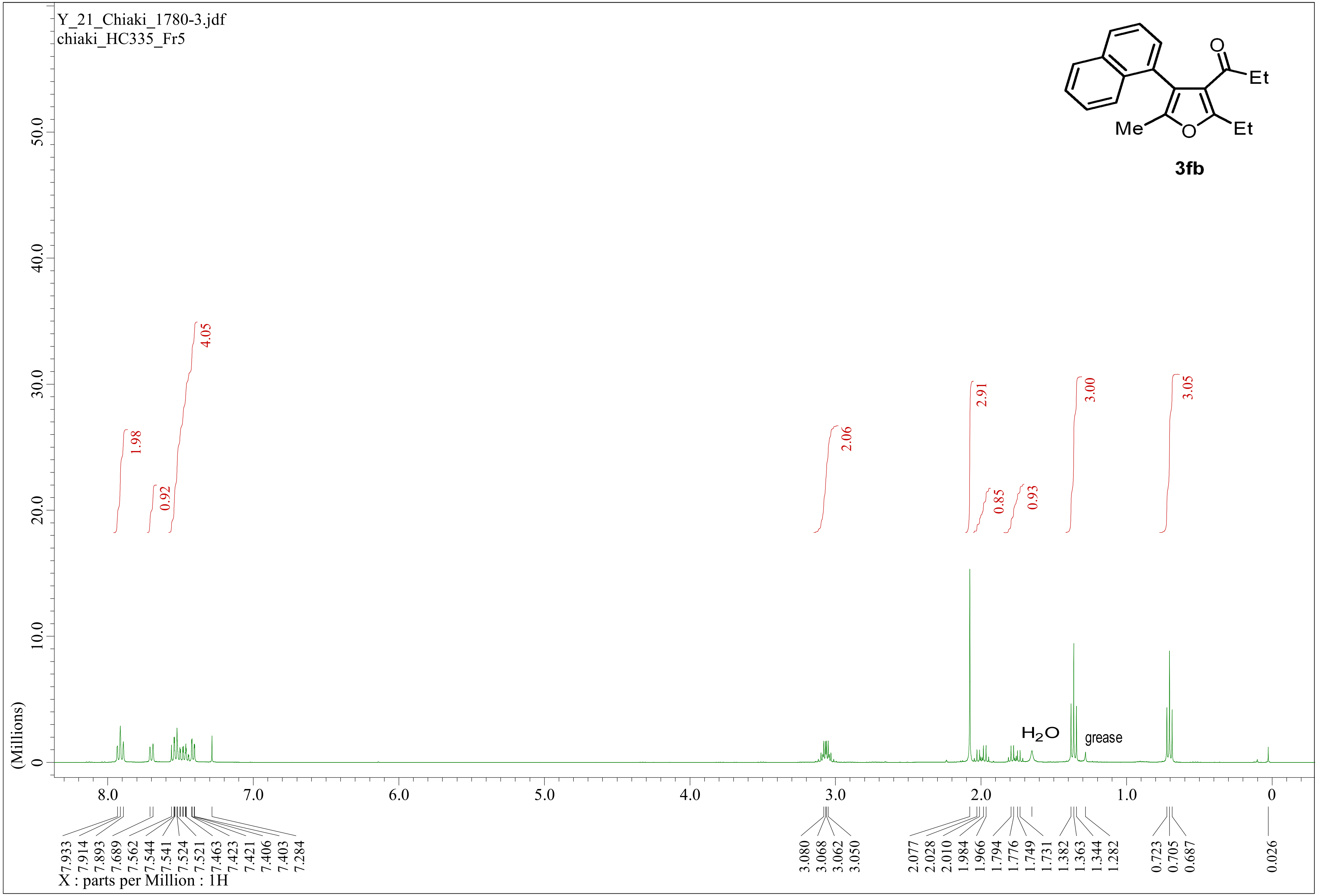

3fb

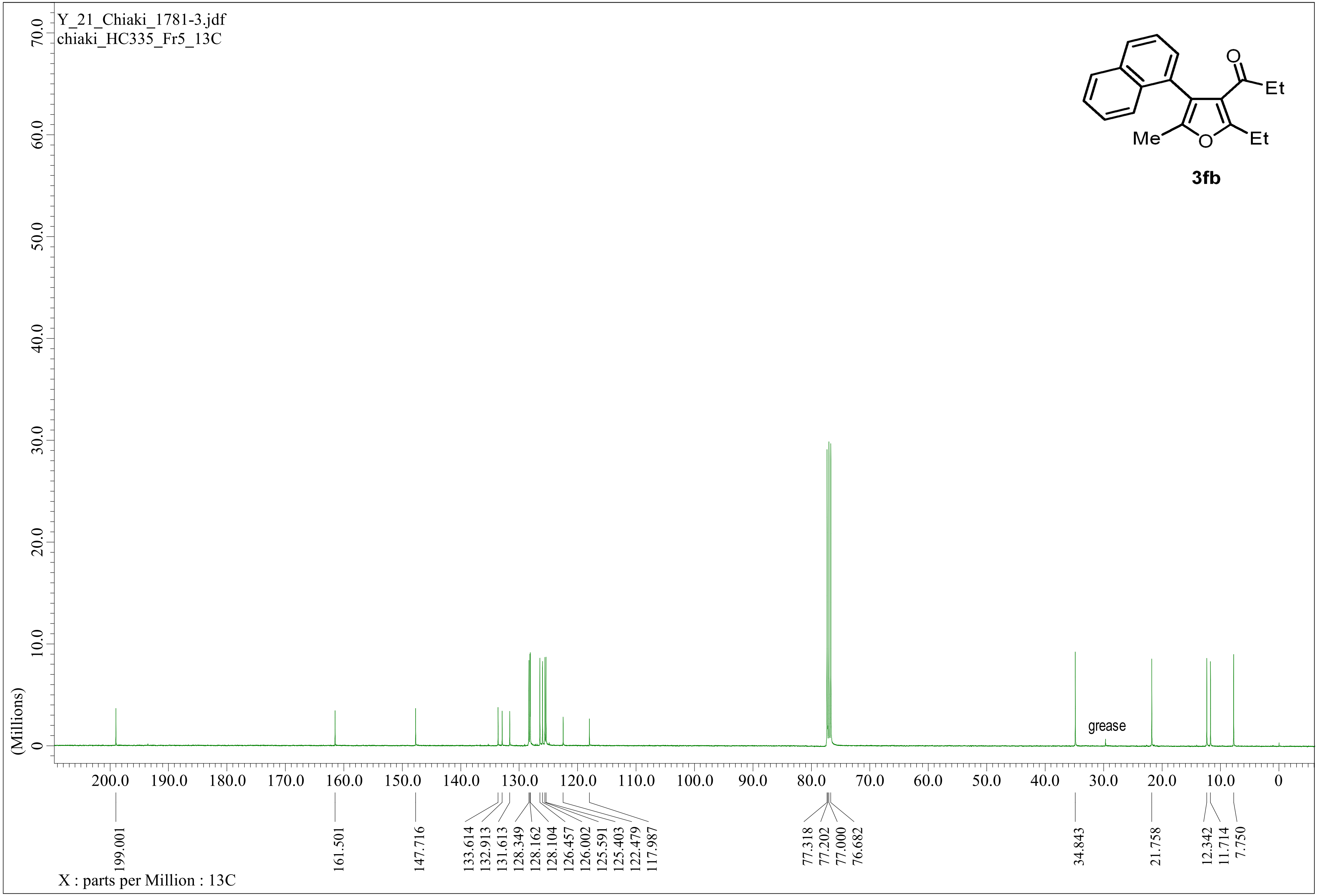

Y_21_Chiaki_1781-3.jdf
chiaki_HC335_Fr5_13C
O
Et
Me
O
Et
3fb
grease
(Millions)
70.0
60.0
50.0
40.0
30.0
20.0
10.0
0
200.0
190.0
180.0
170.0
160.0
150.0
140.0
130.0
120.0
110.0
100.0
90.0
80.0
70.0
60.0
50.0
40.0
30.0
20.0
10.0
0
199.001
161.501
147.716
133.614
132.913
131.613
128.349
128.162
128.104
126.457
126.002
125.591
125.403
122.479
117.987
77.318
77.202
77.000
76.682
34.843
21.758
12.342
11.714
7.750
X : parts per Million : 13C

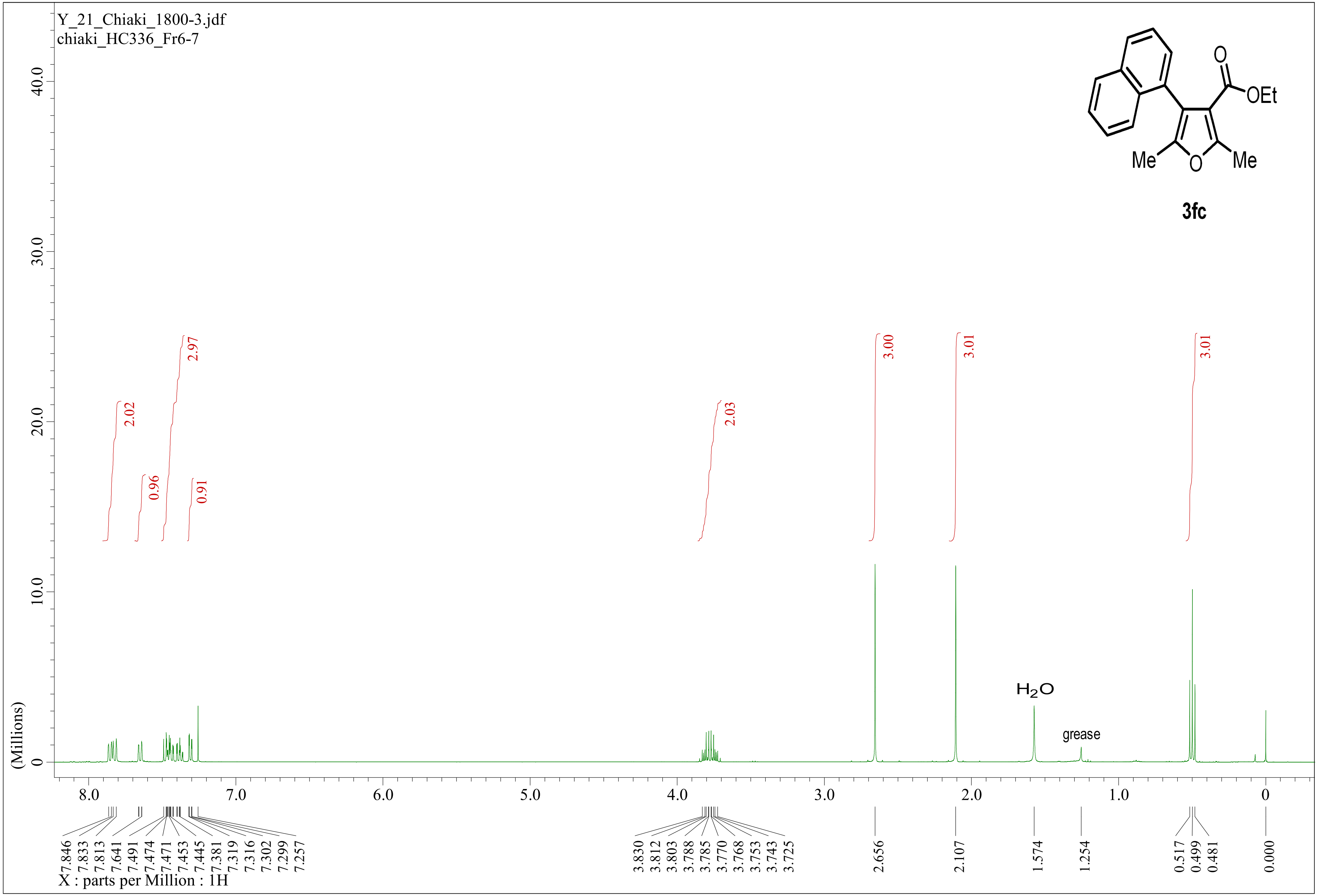

Y_21_Chiaki_1800-3.jdf
chiaki_HC336_Fr6-7
O
OEt
Me
O
Me
3fc
2.02
0.96
2.97
0.91
2.03
3.00
3.01
3.01
H_2O
grease
(Millions)
40.0
30.0
20.0
10.0
0
8.0
7.0
6.0
5.0
4.0
3.0
2.0
1.0
0
7.846
7.833
7.813
7.641
7.491
7.474
7.471
7.453
7.445
7.381
7.319
7.316
7.302
7.299
7.257
3.830
3.812
3.803
3.788
3.785
3.770
3.768
3.753
3.743
3.725
2.656
2.107
1.574
1.254
0.517
0.499
0.481
0.000
X : parts per Million : 1H

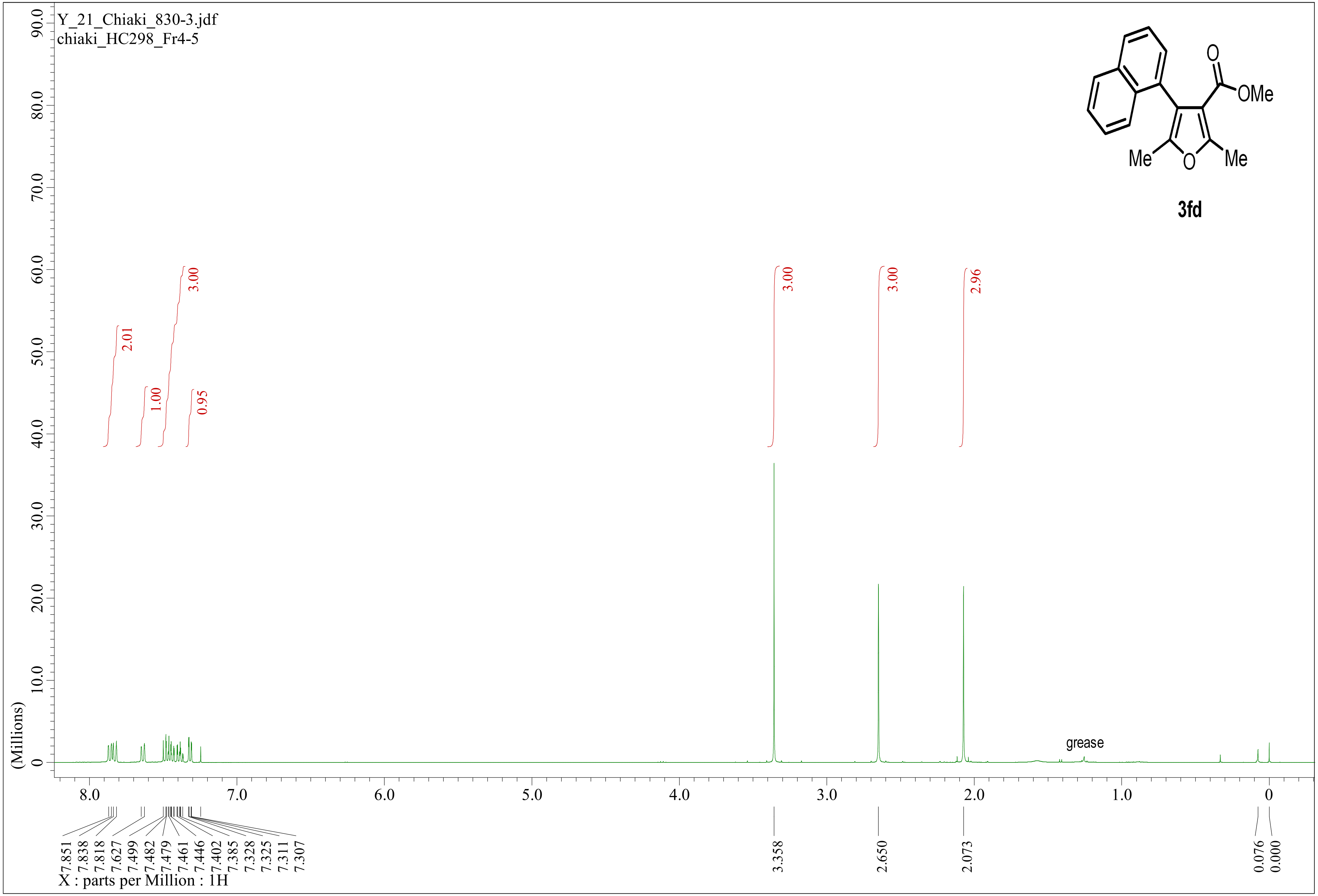
Y_21_Chiaki_830-3.jdf
chiaki_HC298_Fr4-5
OMe
Me
O
Me
3fd
2.01
1.00
3.00
0.95
3.00
3.00
2.96
grease
(Millions)
X : parts per Million : 1H
7.851
7.838
7.818
7.627
7.499
7.482
7.479
7.461
7.446
7.402
7.385
7.328
7.325
7.311
7.307
3.358
2.650
2.073
0.076
0.000

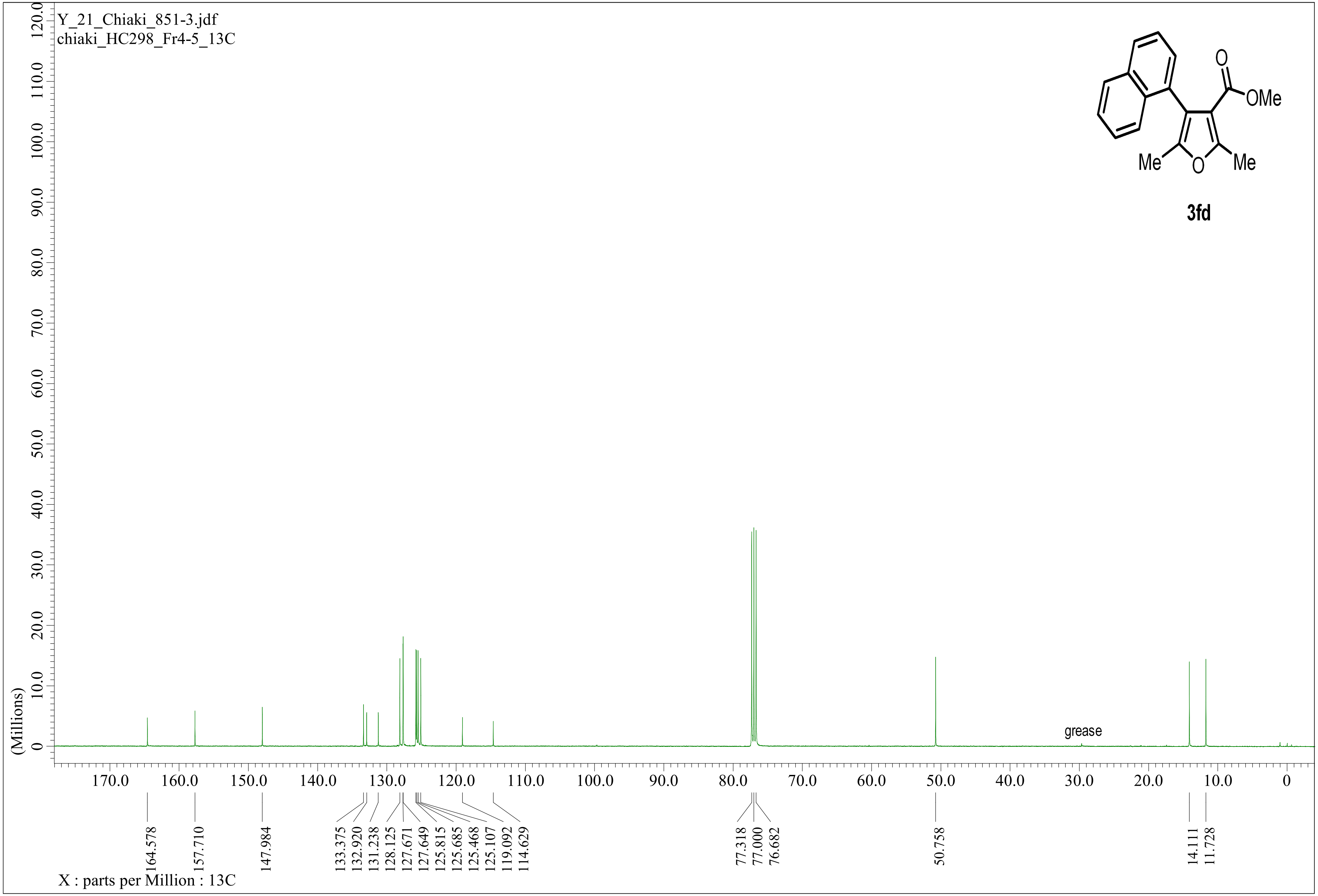

Y_21_Chiaki_851-3.jdf
chiaki_HC298_Fr4-5_13C
OMe
O
Me
O
Me
3fd
(Millions)
0
10.0
20.0
30.0
40.0
50.0
60.0
70.0
80.0
90.0
100.0
110.0
120.0
grease
170.0
160.0
150.0
140.0
130.0
120.0
110.0
100.0
90.0
80.0
70.0
60.0
50.0
40.0
30.0
20.0
10.0
0
164.578
157.710
147.984
133.375
132.920
131.238
128.125
127.671
127.649
125.815
125.685
125.468
125.107
119.092
114.629
77.318
77.000
76.682
50.758
14.111
11.728
X : parts per Million : 13C

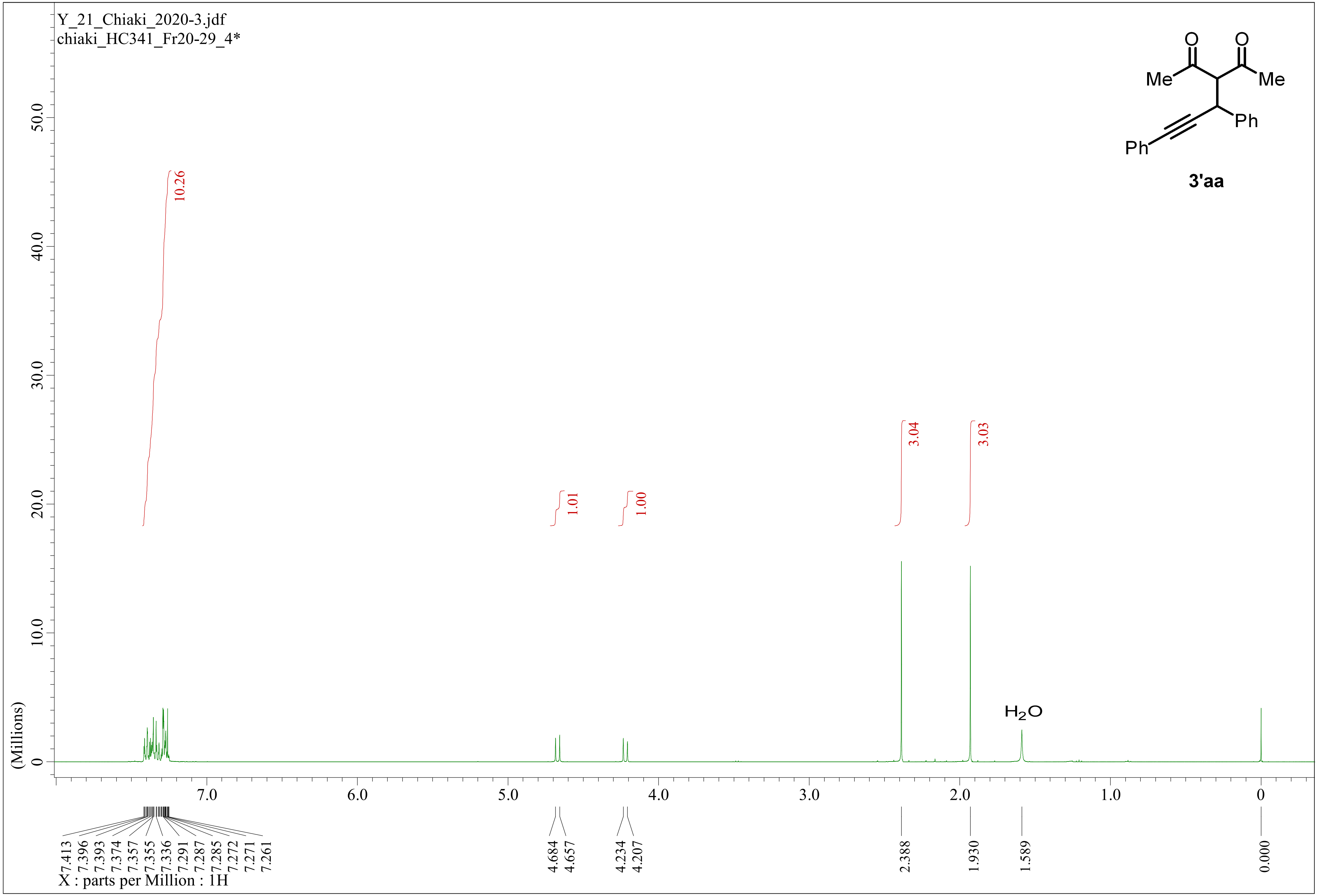
Y_21_Chiaki_2020-3.jdf
chiaki_HC341_Fr20-29_4*
O
O
Me
Me
Ph
Ph
3'aa
10.26
1.01
1.00
3.04
3.03
H2O
(Millions)
0
10.0
20.0
30.0
40.0
50.0
7.0
6.0
5.0
4.0
3.0
2.0
1.0
0
7.413
7.396
7.393
7.374
7.357
7.355
7.336
7.291
7.287
7.285
7.272
7.271
7.261
4.684
4.657
4.234
4.207
2.388
1.930
1.589
0.000
X : parts per Million : 1H

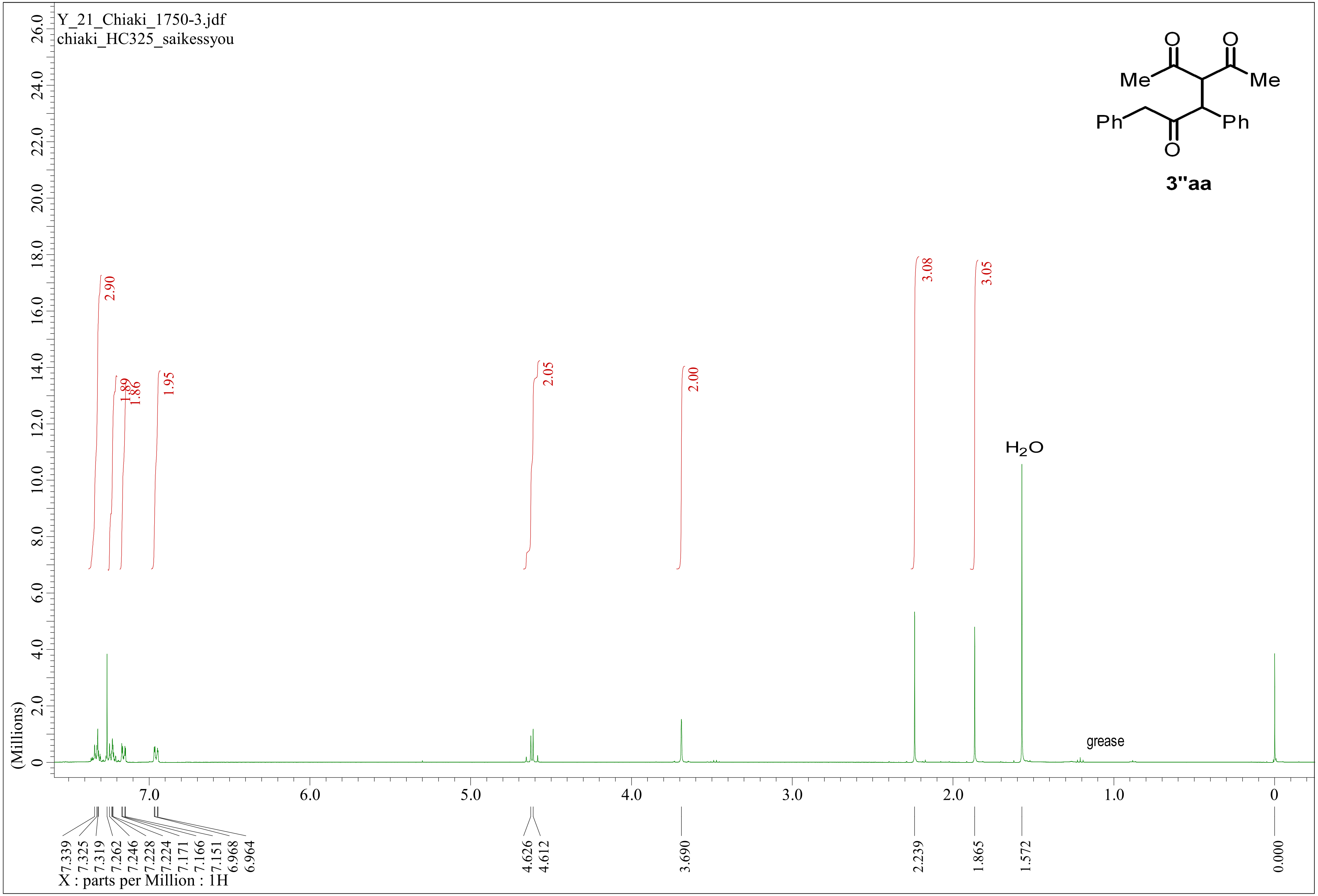
Y_21_Chiaki_1750-3.jdf
chiaki_HC325_saikessyou
3''aa
Me
Me
Ph
Ph
O
O
O
H2O
grease
2.90
1.89
1.86
1.95
2.05
2.00
3.08
3.05
7.339
7.325
7.319
7.262
7.246
7.228
7.224
7.171
7.166
7.151
6.968
6.964
4.626
4.612
3.690
2.239
1.865
1.572
0.000
(Millions)
X : parts per Million : 1H

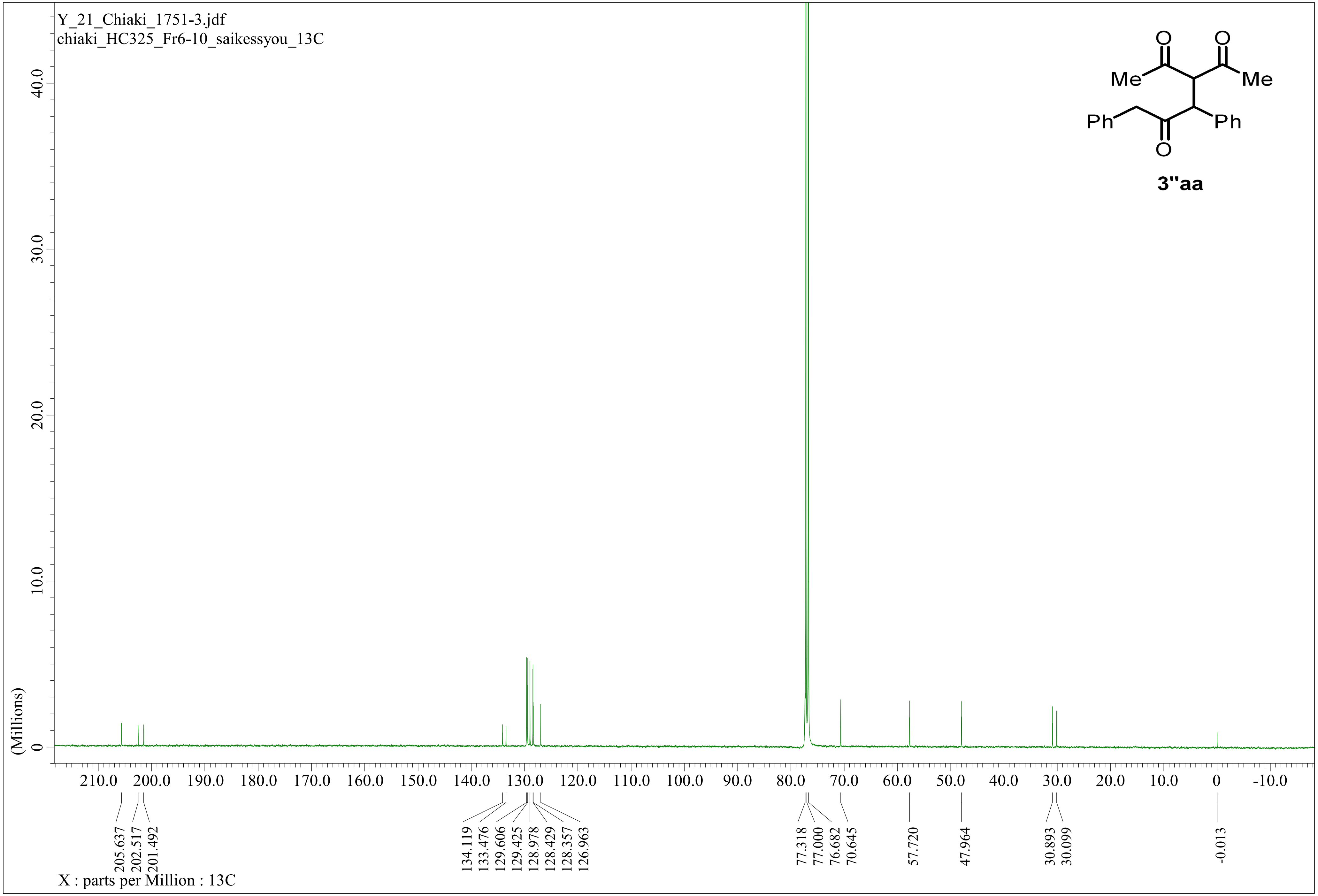
Y_21_Chiaki_1751-3.jdf
chiaki_HC325_Fr6-10_saikessyou_13C
Me
Me
Ph
Ph
O
O
O
3''aa
(Millions)
40.0
30.0
20.0
10.0
0
210.0 200.0 190.0 180.0 170.0 160.0 150.0 140.0 130.0 120.0 110.0 100.0 90.0 80.0 70.0 60.0 50.0 40.0 30.0 20.0 10.0 0 -10.0
205.637
202.517
201.492
134.119
133.476
129.606
129.425
128.978
128.429
128.357
126.963
77.318
77.000
76.682
70.645
57.720
47.964
30.893
30.099
-0.013
X : parts per Million : 13C
